# An atlas of cell-type-specific interactome networks across 44 human tumor types

**DOI:** 10.1186/s13073-024-01303-w

**Published:** 2024-02-12

**Authors:** Zekun Li, Gerui Liu, Xiaoxiao Yang, Meng Shu, Wen Jin, Yang Tong, Xiaochuan Liu, Yuting Wang, Jiapei Yuan, Yang Yang

**Affiliations:** 1 Department of Bioinformatics, School of Basic Medical Sciences, The Province and Ministry Co-Sponsored Collaborative Innovation Center for Medical Epigenetics, Center for Reproductive Medicine, The Second Hospital of Tianjin Medical University, Tianjin Key Laboratory of Inflammatory Biology, Tianjin Medical University, Tianjin, 300070 China; 2 State Key Laboratory of Experimental Hematology, National Clinical Research Center for Blood Diseases, Haihe Laboratory of Cell Ecosystem, Institute of Hematology and Blood Diseases Hospital, Chinese Academy of Medical Sciences and Peking Union Medical College, Tianjin, 300020 China; 3 Tianjin Institutes of Health Science, Tianjin, 301600 China; 4 Department of Pharmacology, School of Basic Medical Sciences, Tianjin Medical University, Tianjin, 300070 China

**Keywords:** Tumor microenvironment, Cell-type-specific, Interactome network, Immune cells

## Abstract

**Background:**

Biological processes are controlled by groups of genes acting in concert. Investigating gene–gene interactions within different cell types can help researchers understand the regulatory mechanisms behind human complex diseases, such as tumors.

**Methods:**

We collected extensive single-cell RNA-seq data from tumors, involving 563 patients with 44 different tumor types. Through our analysis, we identified various cell types in tumors and created an atlas of different immune cell subsets across different tumor types. Using the SCINET method, we reconstructed interactome networks specific to different cell types. Diverse functional data was then integrated to gain biological insights into the networks, including somatic mutation patterns and gene functional annotation. Additionally, genes with prognostic relevance within the networks were also identified. We also examined cell–cell communications to investigate how gene interactions modulate cell–cell interactions.

**Results:**

We developed a data portal called CellNetdb for researchers to study cell-type-specific interactome networks. Our findings indicate that these networks can be used to identify genes with topological specificity in different cell types. We also found that prognostic genes can deconvolved into cell types through analyzing network connectivity. Additionally, we identified commonalities and differences in cell-type-specific networks across different tumor types. Our results suggest that these networks can be used to prioritize risk genes.

**Conclusions:**

This study presented CellNetdb, a comprehensive repository featuring an atlas of cell-type-specific interactome networks across 44 human tumor types. The findings underscore the utility of these networks in delineating the intricacies of tumor microenvironments and advancing the understanding of molecular mechanisms underpinning human tumors.

**Supplementary Information:**

The online version contains supplementary material available at 10.1186/s13073-024-01303-w.

## Background

The cellular processes are achieved by groups of genes acting in concert to shape the cellular responses. Disruption of gene interaction networks could impair cellular functions, leading to diseases such as human tumors [1–3]. Understanding how gene–gene interactions are organized is critical for gaining global insights into the disease mechanisms. Therefore, large efforts have been devoted to building reference interactome networks [4–6]. However, the precise gene interaction networks may be remodeled within their spatiotemporal context [7, 8]. The accumulation of multi-omics data has provided an opportunity to infer accurate tissue-specific gene interaction networks [9, 10]. With the advancement of single-cell RNA sequencing (scRNA-seq) technologies, it is now possible to dissect heterogeneous tissues at a single-cell level [11–13]. The tumor is a heterogeneous mixture comprising of different cell types. scRNA-seq has been widely used to resolve the cellular heterogeneity of tumor microenvironments [11, 14, 15]. Thus, leveraging accumulated scRNA-seq data from human malignancies to examine gene interaction networks within cellular heterogeneity could complement expression-based approaches to elucidating underlying molecular mechanisms.

Therefore, we curated a large-scale compendium of tumor scRNA-seq datasets and built an atlas of cell-type-specific interactome networks across various human tumors. We first collected and uniformly processed scRNA-seq data of 44 different tumor types, totaling approximately 2.2 million single cells from 563 tumor patients. Tumor cells, immune cells, endothelial cells, and other cells were identified using the curated scRNA-seq datasets. Furthermore, by coordinating cell-type annotation across tumor types, a pan-tumor atlas of different immune cell subsets in tumor immune microenvironments (TIMEs) were constructed. Then, we applied the SCINET method to reconstruct interactome networks specific to different cell types and immune cell subsets [16]. SCINET is a reference-guided method for inferring gene interactions from single-cell transcriptome data [16]. Four widely used network resources, including STRING, ConsensusPathDB, HumanNet, and Reactome, were used as the reference interactome [4–6, 17].

We created a data portal, CellNetdb (http://www.bioailab.com:3838/CellNetdb), by designing an interactive user interface that allows users to browse, query, and visualize the cell-type-specific interactome networks. Multifaceted functional data were incorporated to gain more biological insights. The somatic mutation spectra were mapped to the interactome networks. Functional analysis and annotation, including Gene Ontology enrichment and disease enrichment, were also implemented for the networks. Additionally, survival analysis was incorporated to reveal the prognostic relevance of genes implicated in the networks. To speculate the underlying molecular mechanisms modulating ligand-receptor interactions in cell–cell communications, we also inferred cell–cell communications from single-cell transcriptomes and integrated them with the cell-type-specific interactome networks. Furthermore, we found that these cell-type-specific interactome networks enable the distinction of topologically specific genes, whose overall interaction strength is highly cell-type-specific. Through connectivity analysis on different cell-type-specific networks, we can deconvolve prognostic signatures into cell types and found that the prognostic effects of *ITGB1* are linked to CAF-mediated tumor progression. Notably, we have implemented an analysis platform that allows users to prioritize risk genes within cellular heterogeneity. The utility of CellNetdb in prioritizing risk genes have been demonstrated using examples of tumor drivers and T cell exhaustion. We envision that the atlas of cell-type-specific interactome networks in tumors and the CellNetdb portal will help to characterize the tumor microenvironments and reinforce the understanding of molecular mechanisms underlying tumor development and progression.

## Methods

### scRNA-seq data collection and processing

The scRNA-seq data utilized in this study was obtained from a variety of publicly available sources, including the Gene Expression Omnibus (GEO), ArrayExpress, European Nucleotide Archive (ENA), European Genome-phenome Archive (EGA), and Genome Sequence Archive (GSA) (Additional file 1: Table S1). From all curated datasets, the cancer types were categorized based on the original studies, and only malignant samples were included in our analysis (Additional file 1: Table S2 and S3). The raw sequencing data was aligned to the human reference genome (hg38/GRCh38) and UMI count matrices were constructed using *Cell Ranger* (version 5.0.1) [18]. The resulting gene expression matrices were further processed and analyzed using *Seurat* (version 4.1.1) [19]. Quality controls were implemented to filter out cells with low quality based on mitochondrial gene counts, total UMIs, and detected gene counts (percentage of mitochondrial genes > 20%, UMIs < 800 or detected genes counts < 200). The filtered gene expression matrix was then normalized using the *NormalizeData* function. The Seurat CCA was applied to integrated multiple datasets and remove potential batch effects [19]. Dimension reduction and unsupervised clustering were then used to cluster single cells, which were subsequently annotated into distinct cell types using curated marker gene sets (Additional file 1: Table S4). CopyKAT (version 1.0.8) was utilized to assess CNV for each single cell and distinguish malignant cells from normal cells [20]. Malignant cells were identified as cell clusters with significantly abnormal CNV levels.

To construct a pan-tumor TIME atlas of solid tumors, single cells identified as immune cells (myeloid cells, B cells, *CD4*^+^, and *CD8*^+^ T cells) were obtained from the single-cell dataset of each solid tumor type. The Seurat CCA was conducted to integrate immune cells identified as myeloid cells, B cells, *CD4*^+^ T cells, and *CD8*^+^ T cells, respectively, to remove batch effects. Following dimension reduction and unsupervised clustering, the integrated immune cell single-cell transcriptomes were further manually annotated into cell subsets with curated marker gene sets (Additional file 1: Table S5).

### Batch-effect correction and evaluation

As multi-source scRNA-seq data were utilized to construct the single-cell atlas, we conducted an evaluation of the effectiveness of various commonly employed data integration methods, namely BBKNN [21], ComBat [22], Harmony [23], Scanorama [24], scDML [25], scVI [26], and Seurat CCA [19]. Drawing inspiration from previous research, we employed six metrics to assess the tradeoffs between batch integration and clustering performance [25]. These metrics encompassed adjusted rand index (ARI), normalized mutual information (NMI), and average silhouette width for cell type (ASW_celltype) to evaluate clustering performance, as well as inverse Simpson’s index of integration (iLISI), KL divergence of batch mixing (BatchKL), and average silhouette width for batch (ASW_batch) to evaluate the ability to mitigate batch effects. ARI and NMI were employed to quantify clustering accuracy, with higher values indicating greater similarity between the clustering results and true cell types. iLISI was utilized to evaluate the extent of local batch mixing, with higher values indicating superior performance in batch mixing. BatchKL was employed to measure the divergence of batch mixing, with lower values indicating better batch mixing performance. ASW_celltype was used to assess the purity of cell types in clustering, with higher values indicating improved clustering performance. Conversely, ASW_batch was employed to evaluate the extent of global batch mixing after data integration, with lower values indicating better batch-effect correction performance.

Summarizing the benchmarking results of data integration for each tumor type, the overall performance of CCA was found to be either superior or comparable to other methods (Additional file 2: Figure S1A-E). Additionally, the metrics for batch mixing, including distinct studies in our study, were also assessed (Additional file 2: Figure S1F). These observations revealed that CCA exhibited relatively satisfactory performance across all datasets of different tumor types. In evaluating the data integration of pan-tumor TIMEs, CCA demonstrated superior or comparable performance in terms of ARI, NMI, ASW_celltype, and ASW_batch (Additional file 2: Figure S2A-D). Furthermore, the evaluation of batch mixing indicated that CCA achieved the best performance in data mixing in our study (Additional file 2: Figure S2E). Overall, these results highlight the Seurat CCA approach as displaying superior and comparable performance compared to other methods. It exhibits a commendable ability to mix batches while preserving cell type purity, suggesting that CCA is a suitable method for our study.

### Reconstruction of cell-type-specific interactome networks

The SCINET method was employed to reconstruct interactome networks specific to individual cell types [16]. To serve as references, the widely used interactome networks STRING, HumanNet, ConsensusPathDB, and Reactome were downloaded [4–6, 17] (Additional file 1: Table S6). Employing the workflow of SCINET implemented in *ACTIONet*, the cell-type-specific interactome networks were reconstructed for each cell type. Within each tumor type, only cell types with a minimum of 100 cells were utilized for network construction. Leveraging the cell-type annotation, the specificity of genes for each cell type was estimated using the *compute.cluster.feature.specificity* function, followed by the construction of cell-type-specific networks using the *run.SCINET.clusters* function.

### Enrichment analysis of GO terms and disease-associated genes

To facilitate users in acquiring functional insights into the network, we conducted enrichment analysis on Gene Ontology and disease-associated gene sets. The GO gene sets were obtained from the Gene Ontology database (release 2022–06-15, https://release.geneontology.org/2022-06-15, doi: 10.5281/zenodo.6687203), while the disease-associated gene sets were sourced from the DisGeNET database (v7.0) [27]. The statistical significance of the enrichment of genes in GO terms or disease-associated gene sets within each queried local network was determined using the hypergeometric test. The adjusted *P*-values were also calculated using the Benjamini–Hochberg method to correct for multiple testing.

### Integration of somatic mutation

The somatic mutation spectra for each tumor type were obtained from the Catalogue of Somatic Mutations in Cancer (COSMIC) database (v96) [28], which is a comprehensive resource for investigating somatic mutations (Additional file 1: Table S7). Then we assigned the somatic mutation spectra to each gene involved in each cell-type-specific networks across various tumor types and pan-tumor TIMEs.

### Survival analysis

We gathered clinical data from several large-scale cohorts, including The Cancer Genome Atlas (TCGA), Multiple Myeloma Research Foundation (MMRF), and Therapeutically Applicable Research to Generate Effective Treatments (TARGET). Univariate Cox regression analysis was performed to assess the relationship between survival time and gene expression level. Additionally, patients were categorized into two groups based on the median expression level of each gene within cell-type-specific interactome networks. The log-rank test was employed to compare the survival times of high- and low-expression groups, while the Kaplan–Meier curve was utilized to visually represent the observed differences.

### Inference of cell–cell communication

Cell–cell communications were inferred using the R package *CellChat* (version 1.1.3) [29]. A CellChat object was created from the Seurat object using the *createCellChat* function. The CellChat object utilized the *CellChatDB.human* ligand-receptor interaction database. The *computeCommunProb* function was employed to infer the cell–cell communication probability after identifying overexpressed ligands or receptors. The aggregated cell–cell communication network was determined by counting the number of links or summarizing the communication probability. Additionally, the cell–cell communication mediated by the ligand-receptor pairs implicated in the cell-type-specific interactome networks were selected for inclusion in CellNetdb to investigate network genes that modulate cell–cell communications.

### Gene prioritization

We have implemented the random walk with restart (RWR) algorithm to prioritize interested genes based on the cell-type-specific interactome networks. Specifically, the random walk with restart is mathematically defined as follows:

$${p}^{t+1}=\left(1-\gamma \right)W{p}^{t}+{\gamma p}^{0}$$

*W* represents the column-normalized adjacency matrix of the network. The vector ${p}^{t}$ denotes the probability for the random walk to be at node *v* at time *t*, while ${p}^{0}$ is the initial probability vector where only the seed genes have non-zero values. The restart probability, $\gamma$, is set to 0.5. By iteratively repeating the process until the difference between ${p}^{t}$ and ${p}^{t+1}$ falls below 10^−10^, we can numerically approximate the steady-state probability vector. Ultimately, this allows for the ranking of all genes in the network.

### Topological specificity and transcriptional specificity analysis

Topological specificity enables the quantification of the influence of a gene within a cell-type-specific interactome network [16]. In order to evaluate the centrality of genes within a cell-type-specific network, we initially computed the total strength of their local neighbors, represented as *w*^*(celltype)*^*(i)*, for each gene *i*. Subsequently, a random model was constructed to preserve the underlying network topology while uniformly reshuffling the edge weights. This ensemble of random networks allowed us to recompute the strength of interactions, thereby enabling the generation of a distribution of gene neighborhood strengths for each gene. By utilizing the mean *μ*_*R*_^*(celltype)*^*(i)* and standard deviation *σ*_*R*_^*(celltype)*^*(i)* of each distribution, the topological specificity of each gene in a given cell-type-specific network can be defined as follows:

$$topS\left(i\right)= \frac{ {w}^{\left(celltype\right)}\left(i\right)- {\mu }_{R}^{\left(celltype\right)}\left(i\right) }{{\sigma }_{R}^{\left(celltype\right)}\left(i\right)}$$

Transcriptional specificity of genes pertains to their degree of specificity in expression within a particular cell type [16]. To determine this, we employed the gene expression profile to calculate the average expression of various genes in a given cell type *x*_*celltype*_*(i)* and other cell types *x*_*else*_*(i)*. By considering the variance of each group *s*^*2*^_*celltype*_*(i)* and *s*^*2*^_*else*_*(i)*, the transcriptional specificity of each gene in a given cell type can be defined as:

$$tranS\left(i\right)= \frac{{x}_{celltype}\left(i\right)- {x}_{else}\left(i\right) }{\sqrt{\frac{{s}_{celltype}^{2}(i)}{{n}_{celltype}}+\frac{{s}_{else}^{2}(i)}{{n}_{else}}}}$$

### Network similarity evaluation

We employed two distinct metrics, namely shared-edge similarity, and topology similarity, to assess the degree of similarity between networks [30]. Initially, shared nodes were identified between any pair of networks. To quantify the shared-edge similarity, the edges connecting these nodes were extracted from both networks, resulting in the creation of subgraphs for each network. The shared-edge similarity was subsequently determined by calculating the Spearman correlation coefficient between the weights assigned to the shared edges in the respective subgraphs of both networks. To evaluate the topology similarity, the Spearman correlation coefficient was computed for the transformed topological specificity (*topS*_*transf*_) across all shared nodes. The transformation function used for *topS*_*transf*_ was defined as:

$${topS}_{transf}\left(i\right)= \frac{1 }{1+{e}^{-topS(i)}}$$

## Results

### A large-scale single-cell atlas across 44 tumor types

**Fig. 1:**
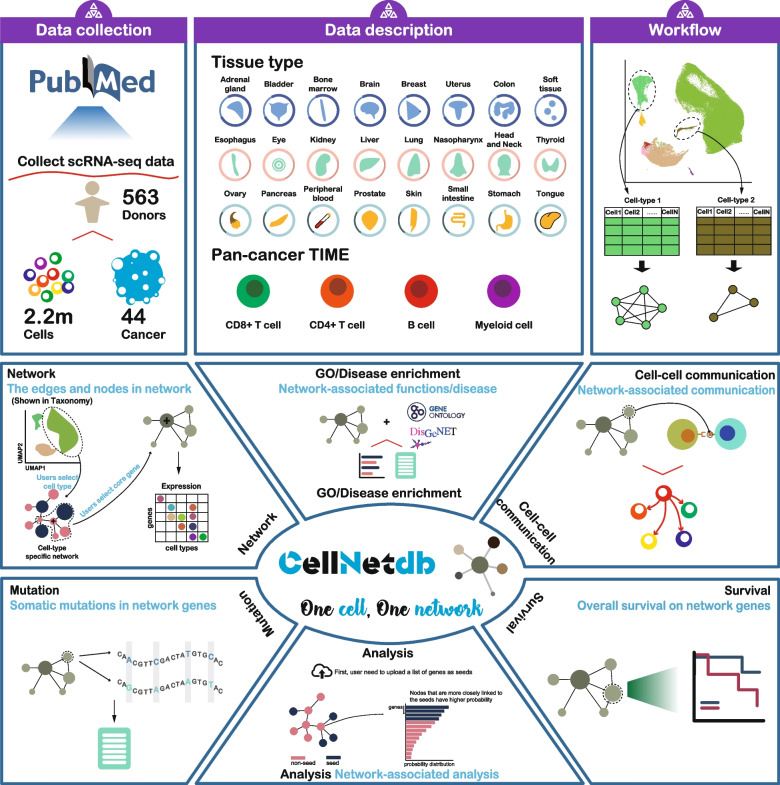
Schematic outline of the overall content is this study. The upper panel depicts the data integration and analysis workflow, including data collection, cell-type annotation, and the reconstruction of cell-type-specific interactome networks. The lower panel shows the function modules of CellNetdb, which provides versatile ways to investigate cell-type-specific interactome networks

To build the atlas of cell-type-specific interactome networks, 563 patients’ scRNA-seq datasets were manually curated through literature searching (Additional file 1: Table S1). We manually filtered, collected, and uniformly processed scRNA-seq data of 44 different tumor types, including 36 solid tumor types and 8 hematological malignancies (Fig. 1, Additional file 2: Figure S3). Following strict quality control and filtration, 1,897,076 cells from solid tumors and 310,965 cells from hematological malignancies were retained. Cell counts ranged from 4590 in gastrointestinal neuroendocrine tumor to 192,889 in colorectal tumor, with a median of 26,299 per tumor type. The sample sizes ranged from 1 in cervical squamous cell carcinoma to 78 in colorectal tumor, with a median of 10 per tumor type (Additional file 2: Figure S3). For each tumor type, we integrated scRNA-seq data from different samples and performed unsupervised clustering to cluster cells into different groups. Then, we assigned cells to distinct cell types, while ensuring expression of canonical cell-type marker genes (Additional file 1: Table S4). A total of 35 different cell types were annotated in solid tumors, and 14 different cell types were annotated in hematological malignancies (Additional file 2: Figure S4).

Furthermore, the annotated *CD4*^+^ T cells, *CD8*^+^ T cells, B cells, and myeloid cells from different solid tumors were integrated with batch-effect correction to define the TIME landscape across solid tumor types. As a result, we built a large-scale pan-tumor atlas of 422,761 immune cells. Subsequently, we performed unsupervised graph-based clustering to identify various cell subsets for each immune cell type (Additional file 2: Figure S5A-D, Additional file 1: Table S5). The *CD4*^+^ T cells were clustered into seven subsets: Trm (*NR4A1*^+^, tissue-resident memory T cells), Th1 (*STAT1*^+^, T helper 1 cells), Th17 (*IL23R*^+^ and *RORC*^+^, T helper 17 cells), Treg (*FOXP3*^+^ and *IL2RA*^+^, regulatory T cells), Tn (*CCR7*^+^, naïve T cells), Tfh (*PDCD1*^+^, follicular T helper cells), and Tem (*GZMK*^+^ and *CCL5*^+^, effector memory T cells) (Additional file 2: Figure S5B). The *CD8*^+^ T cells were grouped into five subsets, including Tc17 (*CCL20*^+^, Type 17 cytotoxic T cells), Temra (*KLRG1*^+^, terminally differentiated effector memory T cells), Tn (*CCR7*^+^, naïve T cells), Tex (*HAVCR2*^+^, exhausted T cells), and Tem (*GZMK*^+^, effector memory T cells) (Additional file 2: Figure S5C). In addition, B cells and Myeloid cells were also partitioned into different subsets (Additional file 2: Figure S5A and S6D).

### An atlas of cell-type-specific interactome networks across different tumor types

Utilizing the constructed single-cell atlas across 44 tumor types, we employed SCINET, a reference-guided method for inferring gene interactions from single-cell transcriptome data, to reconstruct interactome networks specific to different cell types. The reference interactome networks employed in this study include STRING, ConsensusPathDB, HumanNet, and Reactome [4–6, 17] (Additional file 1: Table S6). While STRING, HumanNet, and ConsensusPathDB incorporate physical protein–protein interactions as well as other sorts of interactions, such as genetic interactions, Reactome focuses primarily on reactions, pathways, and biological circuits. Both the number of nodes and number of edges in Reactome are lower than that in other networks (Additional file 2: Figure S6A-B). As a result, the scale of Reactome-guided networks is found to be lower than others when comparing cell-type-specific interactome networks estimated from different reference networks (Additional file 2: Figure S6C-D, Additional file 1: Table S8 and S9).

In addition, we applied the methodology developed by Huang et al. to compare cell-type-specific networks guided by various reference networks [30]. Two metrics, performance and performance gain, were employed to evaluate these networks. Performance was determined by the robust *z*-score of the true AUPRC of the gene set recovery task relative to the background of AUPRCs from the degree-matched null networks. Performance gain was computed as the difference between the AUPRC of a given network and the median AUPRC of its null networks, divided by the median AUPRC of its null networks. Specifically, we focused on the comparison of networks guided by various reference networks using the malignant cell-specific networks as an example. Tumor-associated gene sets for 16 different tumor types were sourced from the DisGeNET database (Additional file 1: Table S10). Performance and performance gain were assessed for the malignant cell-specific network of each tumor type (Additional file 2: Figure S7A-B). A strong correlation between performance and performance gain was observed (Additional file 2: Figure S7C). Our analysis revealed that cell-type-specific networks guided by STRING demonstrated the best overall performance (Additional file 2: Figure S7D).

### CellNetdb access

We have developed CellNetdb, a comprehensive data portal that facilitates the querying and visualization of cell-type-specific interactome networks (Fig. 1). To facilitate the interpretation of these networks, we have implemented versatile functional panels.

**Fig. 2:**
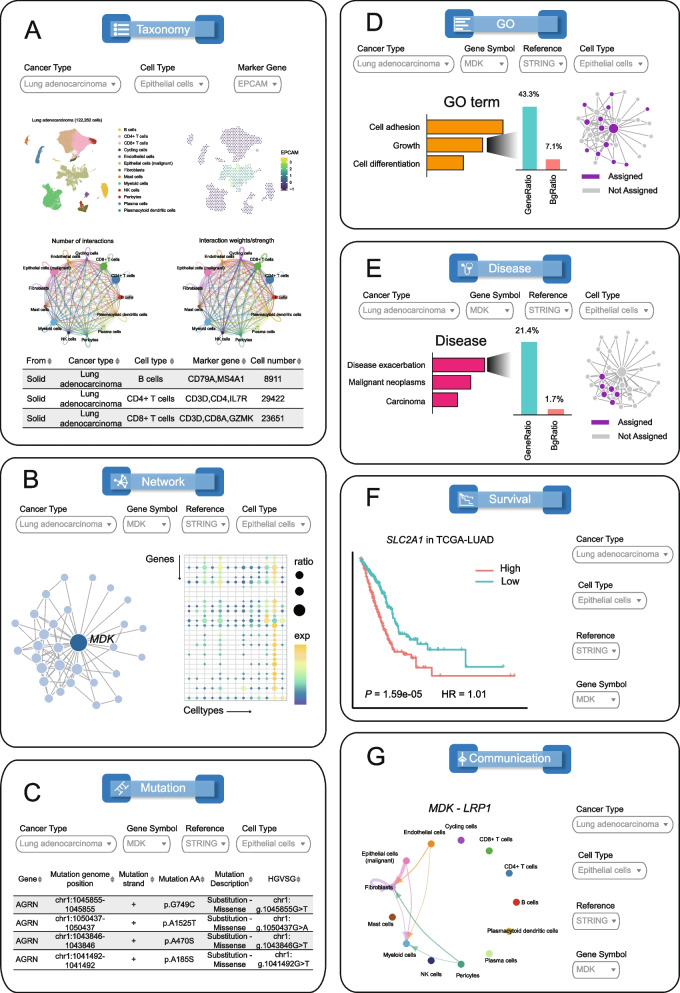
The schematic features in each function module of CellNetdb. **A** The scatterplot represents the UMAP projection of different cell types. The hexbin scatterplot depicts the expression level of *EPCAM* that is a marker gene of epithelial cells. The circle plots show the cell–cell communication between different cell types. Additionally, the number of cells and marker genes for each cell type were described in a tabular form. **B** The graph plot depicts the local network connected to *MDK* gene queried from malignant epithelial cell-specific interactome networks in lung adenocarcinoma. The expression levels of all genes implicated in the local network are shown in the dot-plot heatmap where the color intensity represents the average expression level, and the size of dot represents the percentage of cells. **C** The somatic mutation spectra of each gene implicated in the local network of *MDK* is described in a table. **D, E** GO terms (**D**) and disease gene sets (**E**) enriched for the local network of *MDK*. The bar plots depict the enrichment *P-*values. Those genes of the corresponding GO term (**D**) or disease (**E**) implicated in the local network are labeled in purple. **F** The Kaplan–Meier curve depicts the difference between low- and high-expression level of *SLC2A1* in TCGA lung adenocarcinoma (LUAD) cohort. The log-rank test *P*-value and univariate cox regression hazard ratio are labeled. *SLC2A1* is implicated in the local network of *MDK*. **G** The circle plot shows the cell–cell communication between different cell types mediated by the *MDK*-*LRP1* ligand-receptor pair in lung adenocarcinoma. The line weights represent the strengths of cell–cell communications

The “Taxonomy” page provides an overview of cellular taxonomy based on single-cell transcriptomes (Fig. 1). It also includes information on the cellular taxonomy of pan-tumor immune cell subsets. The use of Uniform Manifold Approximation and Projection (UMAP) allows users to visualize the 2D representation of different cell types and the expression levels of marker genes. Additionally, circle plots are utilized to visualize cell–cell communications based on aggregated signaling pathways. The number of cells for each cell type is presented in a summary table, accessible through the “Summary” panel. As an example, Fig. 2A illustrates the cellular taxonomy of lung adenocarcinoma (LUAD).

The “CellNet” page is designed to investigate cell-type-specific interactome networks with multifaceted function modules (Fig. 1). Users can input gene symbols after selecting the tumor type and cell type to obtain and visualize a local network containing the queried gene and its neighboring genes. Versatile function modules are employed to facilitate the understanding of biological insights. To demonstrate the resource’s usage, we focus on the *MDK* gene in LUAD. *MDK* is frequently upregulated in various human tumors and plays a crucial role in tumor development and progression [31–33]. By selecting lung adenocarcinoma and epithelial cells, users can enter *MDK* to visualize the local network of *MDK* (Fig. 2B). To enhance clarity, the nodes, edges, and edge weights are presented in tabular form. According to the local network, *MDK* exhibits strong connections to *ERBB2* and *ERBB3*. Furthermore, the “Expression” and “Mutation” panels provide gene expression profiles and somatic mutation spectra of *MDK* and its neighbors, respectively (Fig. 2B,C). In terms of functional analysis, the “GO” panel indicates significant enrichment of GO terms related to cell adhesion (GO:0010811, *P*-value = 1.97 × 10^−9^), growth (GO:0040007, *P*-value = 3.56 × 10^−8^), and the ERBB2 signaling pathway (GO:0038128, *P*-value = 1.79 × 10^−6^) within the queried local network (Fig. 2D). Additionally, the local network shows significant associations with disease exacerbation (*P*-value = 4.72 × 10^−6^), malignant neoplasms (*P*-value = 1.12 × 10^−5^), and carcinoma (*P*-value = 4.50 × 10^−5^), as revealed by the “Disease” panel (Fig. 2E). By switching to the “Survival” panel, we observe that the neighboring gene *SLC2A1* is associated with overall survival in LUAD patients (log-rank test *P*-value = 1.59 × 10^−5^) (Fig. 2F).

Finally, the “Communication” panel allows users to investigate how the local network influences cell–cell interactions between different cell types. We found that myeloid cells respond to malignant epithelial cells through the interaction of *MDK* and its receptor *LRP1* (Fig. 2G). This aligns with previous study showing that *MDK* can interact with *LRP1* to promote immunosuppressive macrophage differentiation in ERBB pathway-mutated tumors [34]. Additionally, we discover that the *MDK*-*LRP1* pair is involved in the response of fibroblasts to malignant epithelial cells. Through interrogating the gene–gene interactions in the malignant cell-specific networks and cell–cell interactions between malignant cells and other cells, we identified several other genes may affect the *MDK*-*LRP1*-mediated intercellular crosstalk, such as *ERBB2*, *ERBB3*, *HRAS*, and *ESR1*. The ERBB pathway mutations have been shown to upregulate *MDK* expression in bladder cancer in previous studies. Therefore, CellNetdb can help researchers identify candidate genes that may affect intercellular crosstalk. Overall, CellNetdb aids in unraveling the cellular heterogeneity of gene–gene interactions, enhancing our understanding of the functional roles of genes within the cellular context of tumor development and progression.

### Cell-type-specific network reveals topological specific genes with preferential cell-type influence

Rewiring gene interactions across different cell types can lead to changes in the network topology, which may have varied functional importance depending on the cellular context. Previous studies have demonstrated that constitutive proteins can acquire context-specific effects through tissue-specific interactions [35]. To assess the functional application of cell-type-specific networks in understanding the context-specific role of genes, we utilized a metric called topological specificity (*topS*), introduced by SCINET [16]. This metric allows for the direct quantification of a gene’s influence in a network, beyond what is captured by connectivity and strength alone. The *topS* metric measures the deviation between the observed overall interaction strength of a gene within a specific cell type and the expected strength derived from a random model that preserves the network’s topology while reshuffling the cell-type-specific interaction strengths.

**Fig. 3:**
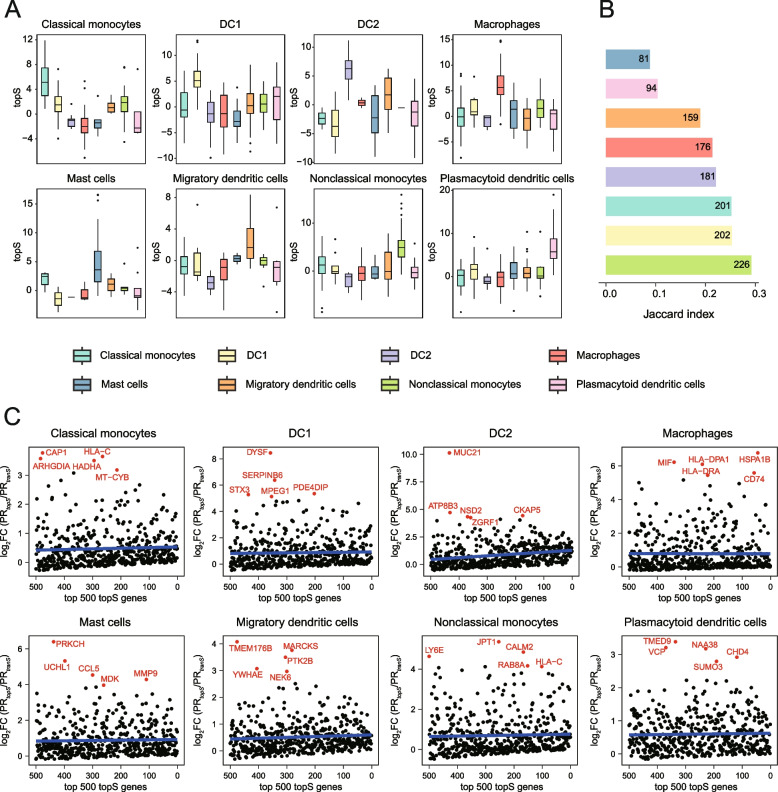
Topologically specific genes with cell-type-specific influence in pan-tumor myeloid cells. **A** The distribution of topologically specific scores (*topS*) is presented for each cell-type-specific network, focusing on canonical marker genes for a specific cell subset in pan-tumor myeloid cells. **B** Comparisons of genes prioritized by *topS* and *tranS* in various cell subsets of pan-tumor myeloid cells is depicted, with Jaccard indices calculated for the top-ranked 500 *topS* and *tranS* genes in each cell subset, and the number of shared genes displayed. **C** Scatterplots illustrating topologically specific genes with broad expression across different cell subsets in pan-tumor myeloid cells, with the best linear regression fit represented by the blue line and the top 5 genes with log2FC(PR_*topS*_/PR_*tranS*_) are highlighted. The log2FC(PR_*topS*_/PR_*tranS*_) represents log2 fold change between percentile ranks of *topS* and *tranS*

To illustrate the application of cell-type-specific networks in understanding the context-specific roles of genes, we focused on myeloid cells in pan-tumor TIMEs as an example. We initially investigated whether known canonical cell-type signature genes play a distinct role in the networks [16] (Additional file 1: Table S11). Our findings revealed that these signature genes exhibited significantly higher *topS* values in the corresponding cell subsets, indicating that the inferred local-interaction topology effectively captures context-relevant biological roles (Fig. 3A). Additionally, we identified top 500 genes for each cell subset based on their *topS* and *tranS* scores, respectively. Notably, there was a moderate concordance between the candidate genes identified by *topS* and *tranS*, even when both transcriptional and topological specificity correlate globally (Fig. 3B, Additional file 2: Figure S8), suggesting that cell-type-specific networks can complement conventional expression analysis.

Furthermore, we identified several sets of genes that exhibited high *topS* scores but low *tranS* scores for each cell subset (Fig. 3C). These genes were found to be expressed in multiple cell subsets but had distinct topological roles across different cell-type-specific networks. Among the top 5 genes with the strongest deviation in *topS* relative to *tranS* per cell subset, we identified genes involved in generic functions such as actin binding (*CAP1*), protein folding (*HSPA1B*), and growth factor activity (*MDK*), as well as genes with more specific functions such as the regulation of macrophage inflammation (*CD74*, a cell membrane high-affinity receptor for MIF). Overall, the cell-type-specific networks enabled us to investigate genes with a preferentially influential role in a cell-type network that cannot be solely explained by their expression pattern.

### Identifying prognosis-associated cell types through network connectivity analysis

**Fig. 4:**
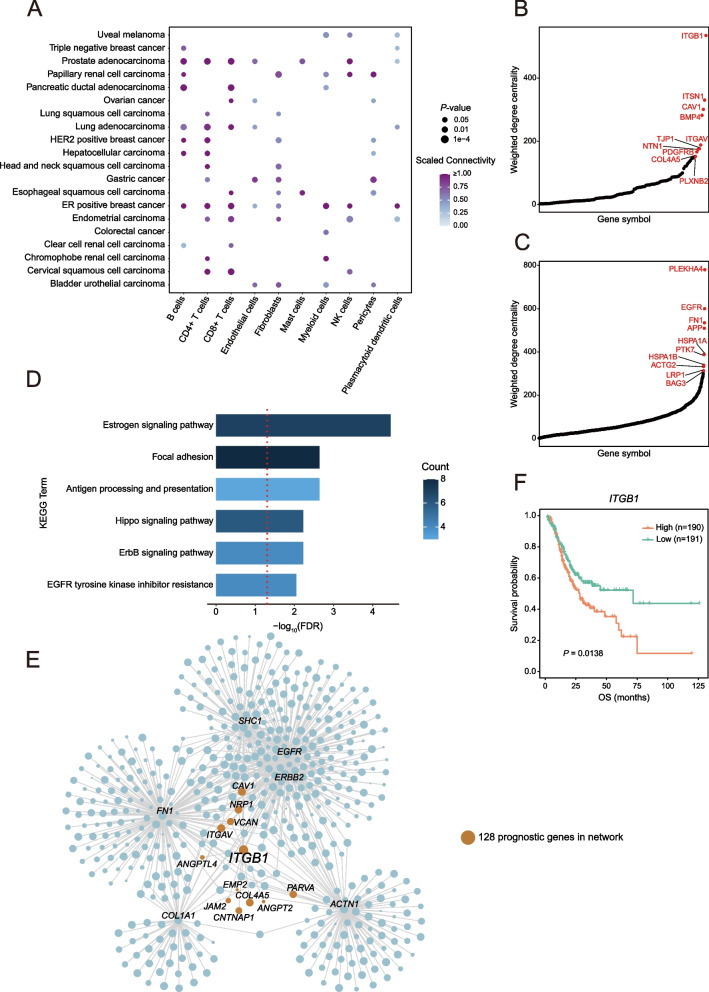
Cell-type deconvolution of cancer prognostic gene signatures. **A** Scaled within-group connectivity of top-ranked cancer prognostic gene sets in different cell-type-specific networks. Cell types presented in more than 10 cancer types were included for analysis. **B** Weighted degree centrality of the 128 prognostic genes in the fibroblast-specific network in gastric cancer, with emphasis on the top 10 genes. **C** Weighted degree centrality of genes neighboring the 128 prognostic genes in the fibroblast-specific network in gastric cancer, highlighting the top 10 genes. **D** Gene set enrichment analysis on the KEGG pathway for the top-ranked 30 genes neighboring the 128 prognostic genes in the fibroblast-specific network in gastric cancer. **E** Visualization of the network of genes neighboring *ITGB1* and several focal adhesion-related genes of the fibroblast-specific network in gastric cancer, with the size of nodes representing the centrality of genes. Brown nodes denote the prognostic genes in the network. **F** Kaplan–Meier plot for gastric cancer patients of TCGA cohort based on the expression of *ITGB1*, showcasing the classification of patients into high-expression and low-expression groups by median value for analysis

The rewiring of molecular networks in diverse cell types with functional relevance can also benefit identifying disease-associated cell types. Previous studies have shown that disease genes tend to form cohesive neighborhoods in the human interactome network [36]. We proposed to deconvolve disease-associated gene sets into individual cell types through evaluating the topological properties of these genes in the cell-type-specific networks, inspired by previous studies [25]. To evaluate the statistical significance of the observed connectivity of a gene set, we employed permutation test approach by generating a null distribution by repeatedly randomly subsampling the same number of genes from the network. Therefore, by applying network connectivity analysis on cell-type-specific networks, we could potentially identify individual cell types associated with tumor patients’ prognosis. With clinical data in TCGA project, we first identified the top 500 prognostic genes based on their expression levels. We ranked these genes using the adjusted *p*-value of the log-rank test, which compares the overall survival of patients with high and low gene expression levels. Through connectivity analysis on different cell-type-specific networks, we found strong network connectivity for prognostic genes in non-cancerous cells, including stromal cells and immune cells (Fig. 4A), indicating that some prognostic genes may function within the non-cancerous cells of the tumor microenvironment.

Next, we focused on the strong connectivity of prognostic genes in the fibroblast-specific interactome networks in gastric cancer. Tumors can activate stromal fibroblast to become cancer-associated fibroblasts (CAFs), which then promote cancer aggressiveness [37]. We identified 128 top prognostic genes in the fibroblast-specific network, with 78 genes closely connected to each other (*P*-value = 0.0028). Among these prognostic genes, several were associated with the metastatic spread of tumors, such as *ITGB1*, *CAV1*, *BMP4*, and *PDGFRB* [38–41], and exhibited a high degree of centrality in the fibroblast-specific network (Fig. 4B). To gain insight into the role of these top-ranked prognostic genes in fibroblasts, we examined the top hub genes directly connected to them (Fig. 4C) and found that top 30 direct neighbor genes were enriched in estrogen signaling pathway and focal adhesion (Fig. 4D). Interestingly, several hub genes directly connected to *ITGB1*, which had the highest degree of centrality among the prognostic genes in the fibroblast-specific networks, included *EGFR*, *FN1*, and *COL1A1* (Fig. 4E). *EGFR* is a member of the ErbB receptor family, known to be involved in CAFs-mediated promotion of tumor invasion and metastasis [42]. *FN1*, encoding fibronectin 1 derived from CAFs, can also promote invasion and metastasis [43]. *COL1A1*, encoding collagen type I alpha 1, has been shown to play a critical role in tumor progression [44]. Besides, the association of *ITGB1* with poor survival has been corroborated by the median expression observed in TCGA-STAD samples (Fig. 4F). Thus, we propose that the prognostic effects of *ITGB1* are linked to CAF-mediated tumor progression. Overall, our findings demonstrate the potential of deconvolving tumor prognostic genes into specific cell types and identifying key target genes within these cell types.

Additionally, tumors which respond well to immune checkpoint blockage (ICB) therapy often have high levels of tumor-infiltrating lymphocytes in their tumor lesions, indicating hot tumors [45]. On the other hand, tumors which do not respond well typically with low T cell infiltration, known as cold tumors [46]. Therefore, we tried to see if the connectivity of cytotoxic gene sets in the *CD8*^+^ T cells can differentiate between hot and cold tumors. We curated 159 cytotoxic genes from the Gene Ontology database and calculated their connectivity in each *CD8*^+^ T cell-specific network for different cancer types. We found that the connectivity of these cytotoxic genes is positively correlated with the proportion of *CD8*^+^ T cells in each tumor sample, serving as a potential indicator of tumor immune profile categorization into hot and cold tumors (Additional file 2: Figure S9). For example, tumor types characterized as hot, such as lung adenocarcinoma, lung squamous cell carcinoma, and head and neck squamous cell carcinoma had high levels of *CD8*^+^ T cells and connectivity of cytotoxic genes. In contrast, cold tumors like glioblastoma and pancreatic ductal adenocarcinoma had low *CD8*^+^ T cell infiltration and connectivity of cytotoxic genes (Additional file 2: Figure S9). Collectively, our findings suggest that the connectivity of cytotoxic genes may serve as a discriminatory factor in distinguishing hot and cold tumors.

### Cell-type-specific interactome network commonality and difference across different tumor types

We conducted a comparative analysis of networks specific to various cell types derived from different solid tumor types. To compare the networks, we employed two metrics based on node topological specificity and interaction strengths, respectively (see “ Methods”) [30]. Our findings suggest that networks specific to the same cell type consistently clustered together, even across different tumor types, indicating that the cellular context primarily shapes the cell-type-specific interactome networks rather than the tumor or tissue types (Additional file 2: Figure S10). Moreover, we observed that the ratio of unique edges to shared edges across tumor types was higher than that of unique nodes to shared nodes, suggesting that networks for the same cell type undergo rewiring in different tissue contexts (Additional file 2: Figure S11). Additionally, we expanded our analysis to include networks specific to various cell types derived from five hematologic malignancies, encompassing all six cell types. Like the analysis of solid tumor types, network similarity analysis revealed a similar clustering pattern (Additional file 2: Figure S12).

**Fig. 5:**
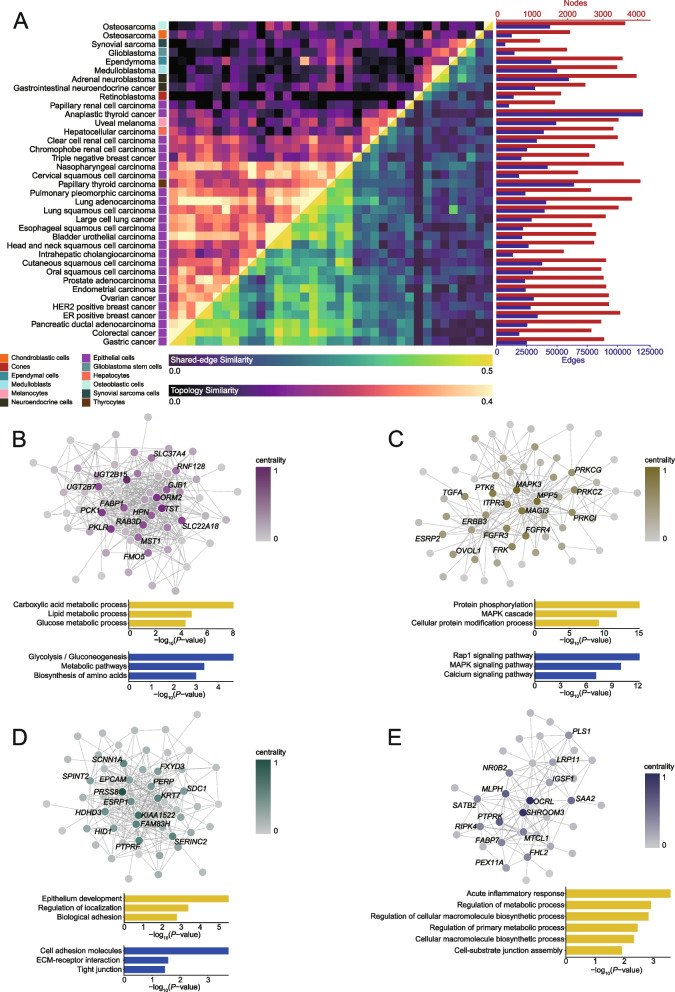
Comparative analysis of interactome networks specific to malignant cells across different tumors. **A** The heatmap depicts the comparison of interactome networks specific to malignant cells identified from different tumor types. The red gradient panel represents the topology similarity estimated from shared nodes’ topological specificity. The green gradient panel represents the edge similarity estimated from shared edges’ interaction strengths. Network sizes are shown by number of nodes (red bars) and number of edges (blue bars). The corresponding tumor types and physiological cell types of different malignant cells are also labeled. **B–E** The graph plots depict four representative core subnetworks identified from the shared network of gastric cancer, colorectal cancer, and pancreatic ductal adenocarcinoma. The centrality of each gene implicated in the subnetwork is labeled in color. The bar plots under each graph plot shows the GO (yellow bars) and KEGG (blue bars) pathway enriched for each core subnetwork

We retrieved and compared malignant cell-specific networks from various solid tumor types, revealing correlation blocks aligned with known physiological conditions of malignancies (Fig. 5A). Notably, malignant epithelial tumors were found to group together, and prominent correlation blocks emerged among different tumor types, such as gastric cancer, colorectal cancer, and pancreatic ductal adenocarcinoma (Fig. 5A). Application of the network community detection method Infomap identified core subnetworks enriched for functional terms related to glycolysis, metabolic processes, protein phosphorylation, MAPK signaling pathway, cell adhesion, and inflammatory response (Fig. 5B–E). The functional interpretation of these core subnetworks aligned with the properties of these tumors, such as hypoxia/Warburg effect, epithelial-to-mesenchymal transition, and tumor-associated inflammation. Hub genes with high centrality, including *PTK6*, *FGFR3*, and *FGFR4*, which have been recognized as therapeutic targets in tumor treatment. Additionally, *MAGI3*, a hub gene connected to them, has been identified as a novel substrate-binding subunit of E3 ligase, suggesting potential regulatory roles of *MAGI3* associated with protein tyrosine kinases that require further verification. Overall, the core subnetworks identified from the shared interactome specific to malignant cells can provide insights into candidate tumor genes and generate testable hypotheses.

Moreover, similar patterns were observed in the comparisons of *CD8*^+^ T cell and *CD4*^+^ T cell-specific networks (Additional file 2: Figure S13 and S14). Several core subnetworks were identified for *CD8*^+^ T cell and *CD4*^+^ T cell-specific networks, respectively. Notably, a core subnetwork enriched for stress response was identified in the shared *CD8*^+^ T cell-specific network (Additional file 2: Figure S13E). These findings align with a recent study indicating that a cellular stress response state of T cells [47]. Collectively, our findings underscore the significance of considering the cellular context.

### Application to prioritizing risk genes

**Fig. 6:**
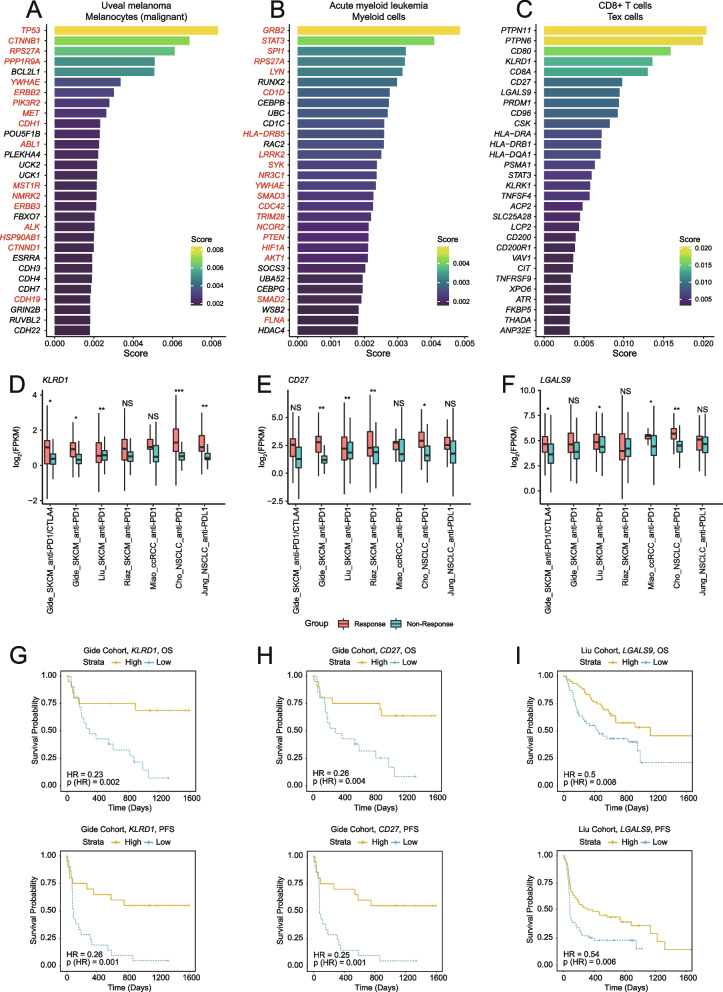
Prioritize risk genes in CellNetdb. **A** Prioritize risk genes using the interactome network specific to malignant melanocytes in uveal melanoma. The bar plot depicts the stationary probability for each top-ranked gene from random walk with restart. The reported cancer driver genes in the NCG 6.0 database are labeled in red. **B** Prioritize risk genes using the interactome network specific to malignant myeloid cells in acute myeloid leukemia. The reported cancer driver genes in the NCG 6.0 database are labeled in red. **C** Prioritize risk genes using the interactome networks specific to exhausted T cells in the pan-tumor TIMEs. **D–F** The expression levels of *KLRD1* (**D**), *CD27* (**E**), and *LGALS9* (**F**) in six ICB therapy cohorts. The boxplots show the difference of their expression levels between ICB therapy response and non-response groups. The cohorts of different tumor types are labeled, including skin cutaneous melanoma (SKCM), clear cell renal cell carcinoma (ccRCC), and non-small cell lung carcinoma (NSCLC). The asterisks represent the degree of significance calculated using Limma (* *P*-value < 0.05, ** *P*-value < 0.01, *** *P*-value < 0.001). **G–I** The Kaplan–Meier curves of overall survival (OS) and progression-free survival (PFS) depict the differences between patients’ groups stratified by the expression level of *KLRD1* (**G**), *CD27* (**H**), and *LGALS9* (**I**), respectively

The identification of genes involved in cancer progression presents a significant challenge with critical implications for understanding biological processes. The use of cell-type-specific networks provides a promising approach for extending gene prioritization within the context of specific cell types. Several network-based prioritization methods have been developed to rank disease-associated genes, prompting the need to determine the most suitable method. To address this, a benchmarking analysis was conducted to evaluate the performance of four representative methods—RWR [48], GenePanda [49], Node2Vec [50], and DIAMOnD [51]. Utilizing genes annotated in the Cancer Gene Census (CGC) as seed genes [52], the prioritization of risk genes was performed using malignant cell-specific networks from six different tumor types. Subsequently, the NCG 6.0 database was used as a benchmark to assess the performance of each method [53]. The results indicated that the RWR outperformed the others in prioritizing risk genes by leveraging biological networks (Additional file 2: Figure S15). As a result, the RWR method has been implemented into CellNetdb for prioritizing risk genes within the context of cell-type-specific interactome networks. The platform allows users to upload a gene list as seed genes for prioritizing risk genes within cell-type-specific networks. Case studies in uveal melanoma and acute myeloid leukemia were provided to demonstrate the utility of this platform, revealing a high proportion of candidate genes annotated as cancer drivers (Fig. 6A,B and Additional file 2: Figure S16 and S17). Additionally, genes not reported by NCG may also have roles in tumors, as demonstrated by the examples of *BCL2L1*, *PLEKHA4*, and *RUNX2* [54–56].

Additionally, users can use interactome networks specific to different immune cell subsets to prioritize risk genes. For example, inputting a list of genes associated with T cell exhaustion as seed genes will return a list of candidate genes prioritized using the networks specific to *CD8*^+^ exhausted T cells (Fig. 6C and Additional file 2: Figure S18). Some of the top-ranked genes, such as *PTPN11* (*SHP2*) and *PTPN6* (*SHP1*), have been linked to T cell exhaustion [57–61]. To validate additional top-ranked candidate genes, six ICB therapy-related cohorts were collected (Additional file 1: Table S12), totaling 399 samples treated with CTLA-4 or PD1/PD-L1 inhibitors in skin cutaneous melanoma (SKCM), clear cell renal cell carcinoma (ccRCC), and non-small cell lung carcinoma (NSCLC) [62–67]. Comparative analysis revealed that the expression levels of several top-ranked genes differ among patients based on their response to ICB therapy (Fig. 6D–F, Additional file 2: Figure S19). For example, the *KLRD1* gene is significantly downregulated in pretreatment patients of non-response groups compared to response groups. Additionally, high-expression groups of *KLRD1*, *CD27*, and *LAGLAS9* significantly outlive low-expression groups in terms of both progression-free survival (PFS) and overall survival (OS) (Fig. 6G–I). These findings suggest that the top-ranked genes in the prioritization analysis may be involved in T cell exhaustion, potentially affecting the effectiveness of ICB treatment. This indicates that interactome networks specific to different immune cell subsets may be a valuable resource for prioritizing important genes involved in cancer immunity.

## Discussion

In this study, we provided a high-resolution view of tumor microenvironments across 44 different tumor types. Given the heterogeneity of intratumoral immune cells, we have also undertaken the creation of a pan-tumor single-cell atlas of the tumor immune microenvironments, which span 36 solid tumor types, thereby supplementing prior research. Our analyses have facilitated the construction of a compendium of cell-type-specific interactome networks in tumors, by employing four reference interactomes of high performance.

In the realm of network biology, our study highlights the importance of topological specificity in accurately quantifying a gene’s influence within a cell-type-specific network. This attribute holds promise for identifying genes with broad expression but condition-specific interactions in future studies. Furthermore, we have demonstrated applications of cell-type-specific networks in investigating the cell-type specificity of disease-associated genes. Firstly, disease genes can be deconvolved into cell types based on network connectivity across cell-type-specific networks. For instance, deconvolution of gastric cancer prognostic signatures revealed high connectivity in non-cancerous cells, where stromal fibroblasts can be activated by tumors to differentiate into CAFs, promoting cancer aggressiveness. We identified *ITGB1*, one of the hub genes, as having prognostic effects linked to CAF-mediated tumor progression. Future efforts may involve expanding the deconvolution of disease gene sets for all cell types of each tissue and identifying pivotal target genes within these cell types. Secondly, we could identify potential cancer drivers by using well-known cancer drivers as seed genes to prioritize genes in malignant cell-specific networks. Furthermore, we developed CellNetdb, a web portal that facilitates interactive exploration of cell-type-specific interactome networks. Diverse functionalities were incorporated to obtain comprehensive biological insights into cell-type-specific interactome networks. Users can query genes of interest to obtain a local network with multifaceted functional data, enabling them to generate testable hypotheses. Additionally, we have implemented an analysis platform in CellNetdb for prioritizing risk genes.

Nevertheless, this study has some limitations, particularly in comparison to previous research focused on specific tumor types. The pan-tumor single-cell atlas in this study offers relatively coarse resolutions for cell types or cell states, potentially hindering the inference of cell-type-specific interactome networks due to the noisy and sparse nature of single-cell transcriptome data. Additionally, while the reference-guided approach employed in this study allowed for an accurate reconstruction of cell-type-specific networks, it limited the discovery of novel unique interactions specific to the cell type. Addressing these limitations will require the collection of more tumor scRNA-seq data and additional analyses in future research.

## Conclusions

In this study, we introduce CellNetdb, a comprehensive database containing a large-scale atlas of cell-type-specific interactome networks within tumor microenvironments. We created these networks by analyzing single-cell RNA-seq data from 563 patients, which included over two million cells from 44 different tumor types. The database offers various functionalities designed to provide in-depth biological insights. We also showcased the practical application of the cell-type-specific networks, including the identification of topologically specific genes, cell-type deconvolution of prognostic genes, and the prioritization of risk genes. We believe that CellNetdb has the potential to be a valuable resource for exploring candidate genes and generating testable hypotheses, ultimately contributing to a deeper understanding of tumor microenvironments and the advancement of precision oncology.

### Supplementary Information

**Additional file 1: Table S1.** The collection of scRNA-seq data used in this study. **Table S2.** The scRNA-seq datasets of solid tumor samples used in this study. **Table S3.** The scRNA-seq datasets of hematologic malignancy samples used in this study. **Table S4.** Curated marker genes for different cell types. **Table S5.** Curated marker genes for different immune cell subsets. **Table S6.** Information of four reference networks used in this study. **Table S7.** Curated COSMIC somatic mutation data for each solid tumor type. **Table S8.** Number of nodes and edges of cell-type-specific networks in each tumor type. **Table S9.** Number of nodes and edges of cell-subset-specific networks in pan-tumor TIMEs. **Table S10.** DisGeNET disease gene sets used in network performance evaluation. **Table S11.** Canonical marker genes for 8 cell subsets in myeloid cells of pan-tumor TIMEs. **Table S12.** The data collection of six ICB therapy cohorts.

**Additional file 2: Figure S1.** Evaluation of the performance of different batch correction methods in each tumor type. **Figure S2.** Evaluation of the performance of different batch correction methods in pan-tumor TIME integration. **Figure S3.** Summary of the single-cell atlas across 44 tumor types. **Figure S4.** Cell types annotated in the single-cell atlas. **Figure S5.** The expression levels of marker genes in different immune cell subsets. **Figure S6.** Statistics of cell-type-specific interactome networks guided by four different reference networks. **Figure S7.** Network performance of recovering DisGeNET disease gene sets. **Figure S8.** Comparison of topological specificity and transcriptional specificity scores in myeloid cells of pan-tumor TIMEs. **Figure S9.** Connectivity of cytotoxic gene sets within *CD8*^+^ T cell-specific networks across different tumor types. **Figure S10.** Network similarity across 17 solid tumor types. **Figure S11.** Comparison of *CD8*^+^ T cells networks across 17 solid tumors. **Figure S12.** Network similarity across five hematologic malignancies. **Figure S13.** Comparative analysis of interactome networks specific to *CD8*^+^ T cells. **Figure S14.** Comparative analysis of interactome networks specific to *CD4*^+^ T cells. **Figure S15.** Comparing four network-based gene prioritization methods. **Figure S16.** Prioritize risk genes in uveal melanoma. **Figure S17.** Prioritize risk genes in acute myeloid leukemia. **Figure S18.** Prioritize risk genes for T cell exhaustion. **Figure S19.** The expression levels of risk genes prioritized for T cell exhaustion in ICB cohorts.

## Data Availability

CellNetdb is publicly and freely available at http://www.bioailab.com:3838/CellNetdb to all users without any login or registration restrictions. All public scRNA-seq and RNA-seq datasets used in this study are available in Additional file 1: Table S1 and Table S12. The analysis code is available at GitHub (https://github.com/YY-TMU/CellNetdb) [68].

## Abbreviations

TIME: Tumor immune microenvironment
UMI: Unique molecular identifier
GO: Gene Ontology
ARI: Adjusted rand index
NMI: Normalized mutual information
ASW_celltype: Average silhouette width for cell type
iLISI: Inverse Simpson’s index of integration
BatchKL: KL divergence of batch mixing
ASW_batch: Average silhouette width for batch
TCGA: The Cancer Genome Atlas
MMRF: Multiple Myeloma Research Foundation
TARGET: Therapeutically Applicable Research to Generate Effective Treatments
RWR: Random walk with restart
UMAP: Uniform Manifold Approximation and Projection
LUAD: Lung adenocarcinoma
ICB: Immune checkpoint blockade
CGC: Cancer Gene Census
NCG: The Network of Cancer Genes
AML: Acute myeloid leukemia
SKCM: Skin cutaneous melanoma
ccRCC: Clear cell renal cell carcinoma
NSCLC: Non-small lung carcinoma
PFS: Progression-free survival
OS: Overall survival
