# Supplementary material for: An atlas of cell-type-specific interactome networks across 44 human tumor types
Source: Genome Med. 2024 Feb 12;16:30. doi: 10.1186/s13073-024-01303-w (PMC10860273; doi:10.1186/s13073-024-01303-w)
Supplement: Supplementary file 2 — Additional file 2: Figure S1. Evaluation of the performance of different batch correction methods in each tumor type. Figure S2. Evaluation of the performance of different batch correction methods in pan-tumor TIME integration. Figure S3. Summary of the single-cell atlas across 44 tumor types. Figure S4. Cell types annotated in the single-cell atlas. Figure S5. The expression levels of marker genes in different immune cell subsets. Figure S6. Statistics of cell-type-specific interactome networks guided by four different reference networks. Figure S7. Network performance of recovering DisGeNET disease gene sets. Figure S8. Comparison of topological specificity and transcriptional specificity scores in myeloid cells of pan-tumor TIMEs. Figure S9. Connectivity of cytotoxic gene sets within CD8+ T cell-specific networks across different tumor types. Figure S10. Network similarity across 17 solid tumor types. Figure S11. Comparison of CD8+ T cells networks across 17 solid tumors. Figure S12. Network similarity across five hematologic malignancies. Figure S13. Comparative analysis of interactome networks specific to CD8+ T cells. Figure S14. Comparative analysis of interactome networks specific to CD4+ T cells. Figure S15. Comparing four network-based gene prioritization methods. Figure S16. Prioritize risk genes in uveal melanoma. Figure S17. Prioritize risk genes in acute myeloid leukemia. Figure S18. Prioritize risk genes for T cell exhaustion. Figure S19. The expression levels of risk genes prioritized for T cell exhaustion in ICB cohorts. [file 13073_2024_1303_MOESM2_ESM.pdf]

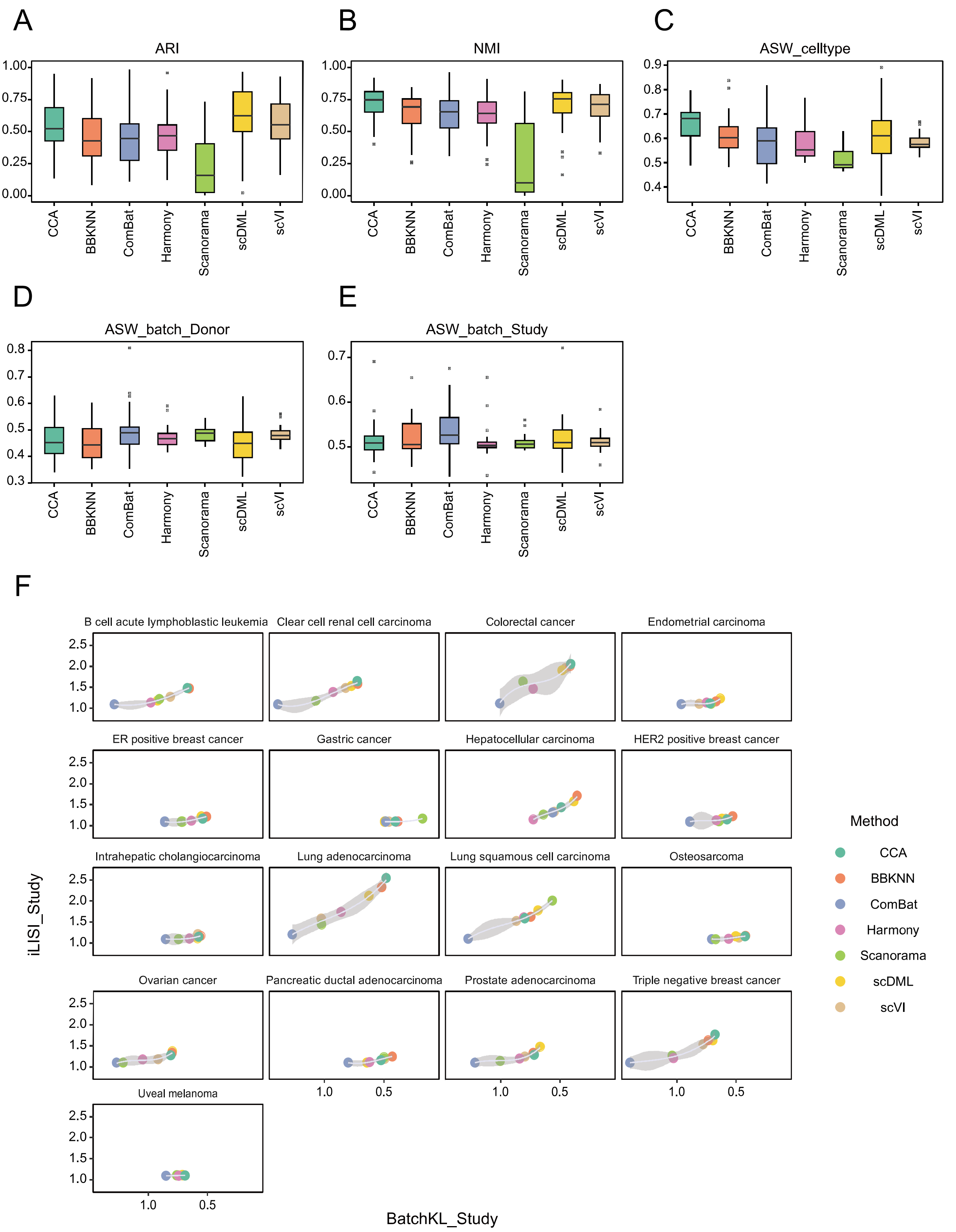

Figure S1. Evaluation of the performance of different batch correction methods in each tumor type.

(A-E) Box plots show the value of **(A)** ARI, **(B)** NMI, **(C)** ASW_celltype, **(D)** ASW_batch_Donor **and (E)** ASW_batch_Study for seven different methods in 41 tumor types.

(F) Scatter plots show the value of BatchKL_Study (X-axis) and iLISI_Study (Y-axis) across 17 distinct tumor types incorporating data from different studies. Points situated closer to the upper right of the plot correspond to superior performance. The error bands depicted in the plots signifies the 95% confidence interval surrounding the smoothed curve, which was achieved through B-spline smoothing with a degree of 3.

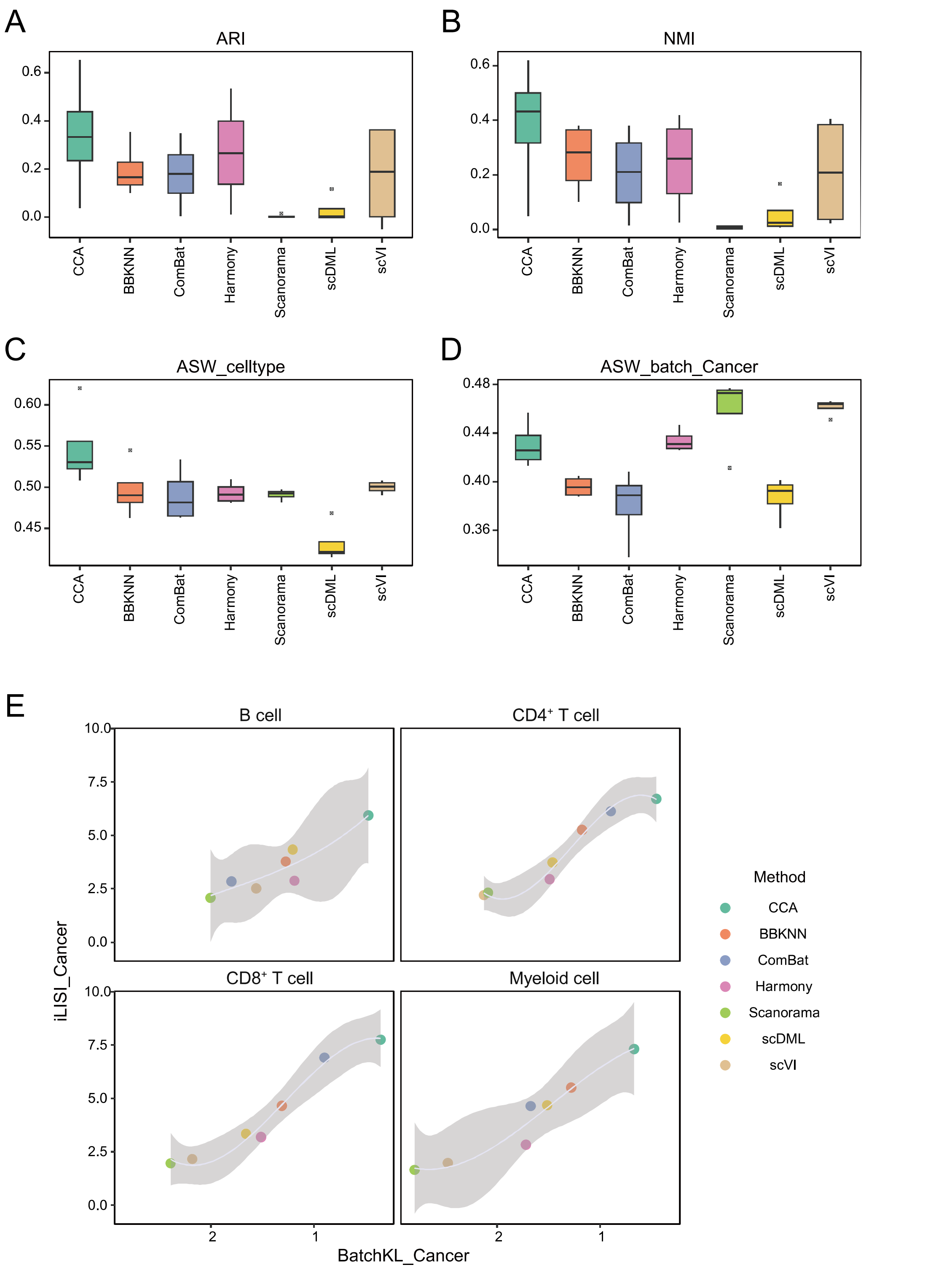

Figure S2. Evaluation of the performance of different batch correction methods in pan-tumor TIME integration.

(A-D) Box plots show the value of **(A)** ARI, **(B)** NMI, **(C)** ASW_celltype and **(D)** ASW_batch_Cancer for seven different methods in the pan-tumor TIME integration.

(E) Scatter plots show the value of BatchKL_Cancer (X-axis) and iLISI_Cancer (Y-axis) across

four different tumor infiltrating immune cells in pan-tumor TIME integration. Points situated closer to the upper right of the plot correspond to superior performance. The error bands depicted in the plots signifies the 95% confidence interval surrounding the smoothed curve, which was achieved through B-spline smoothing with a degree of 3.

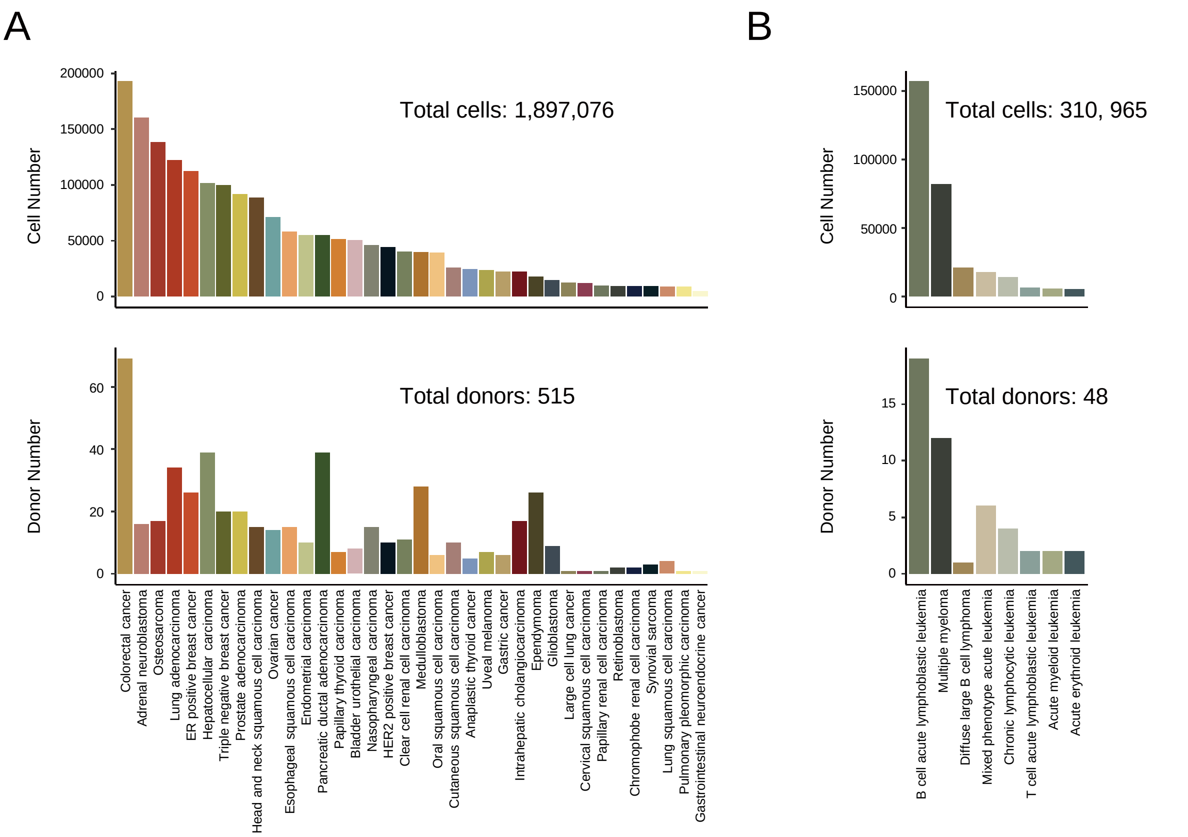

Figure S3. Summary of the single-cell atlas across 44 tumor types.

(A) Summary of the single-cell atlas across 36 solid tumor types. The upper panel depicts number of cells identified for each tumor type. The lower panel depicts the number of patients collected for each tumor type.

(B) Summary of the single-cell atlas across 8 hematological malignancies. The upper panel depicts number of cells identified for each tumor type. The lower panel depicts the number of patients collected for each tumor type.

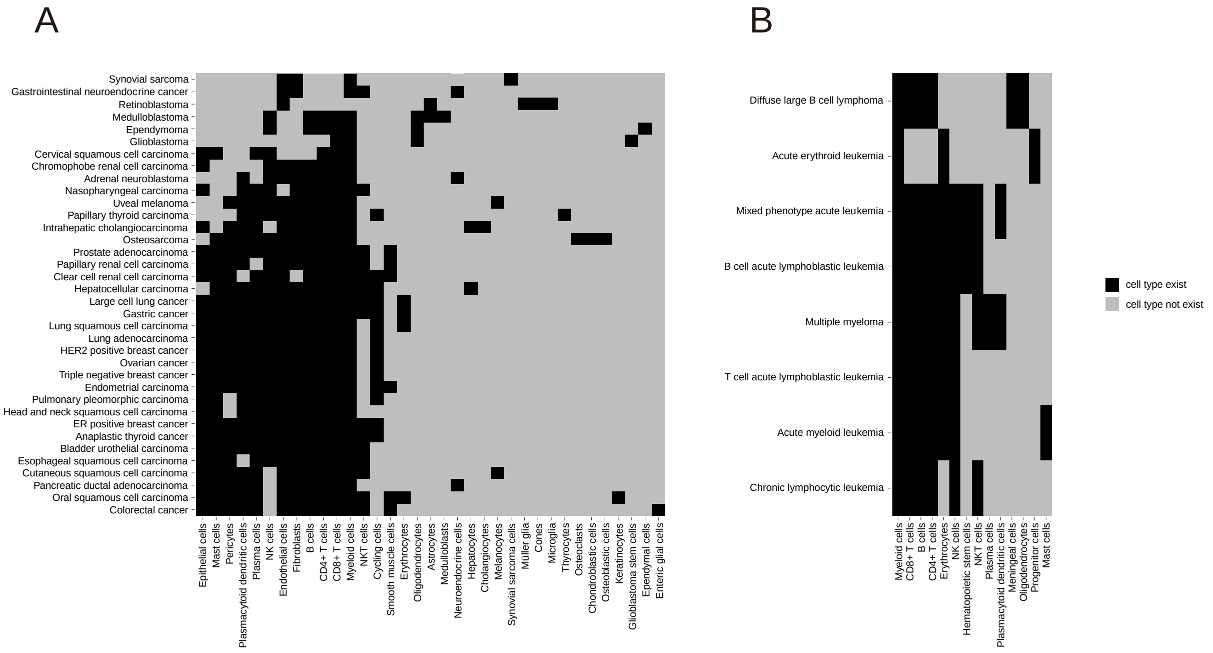

Figure S4. Cell types annotated in the single-cell atlas.

The heatmaps show the cell type annotated in all tumor types, including **(A)** solid tumors and **(B)** hematological malignancies.

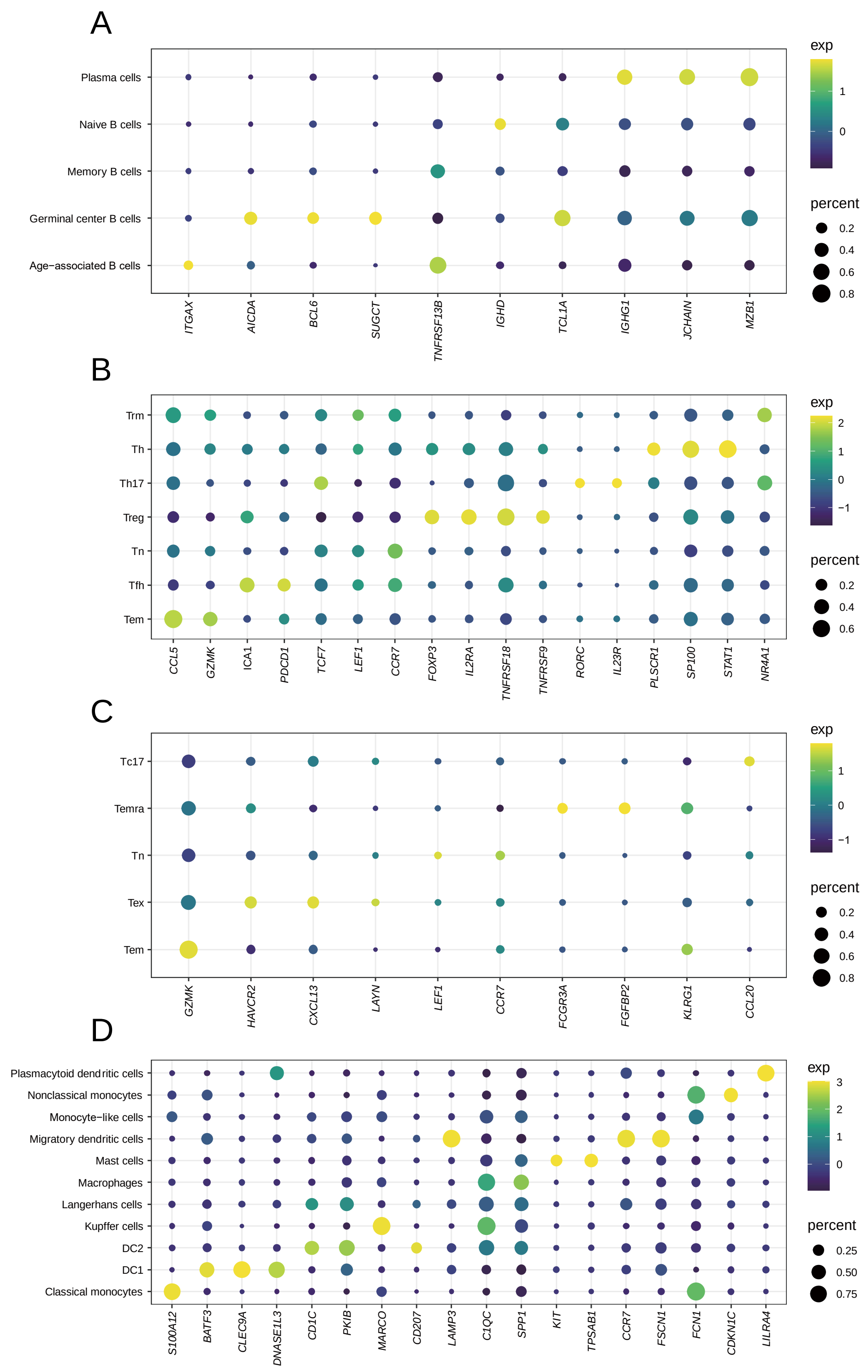

Figure S5. The expression levels of marker genes in different immune cell subsets.

The dot-plot heatmaps depict the expression levels of marker genes in different immune cell subsets identified from B cells **(A)**, *CD4*⁺ T cells **(B)**, *CD8*⁺ T cells **(C)**, and myeloid cells **(D)**. The color intensity represents the average gene expression level, and the size of dot

represents the percentage of cell within each immune cell subsets expressing the marker gene.

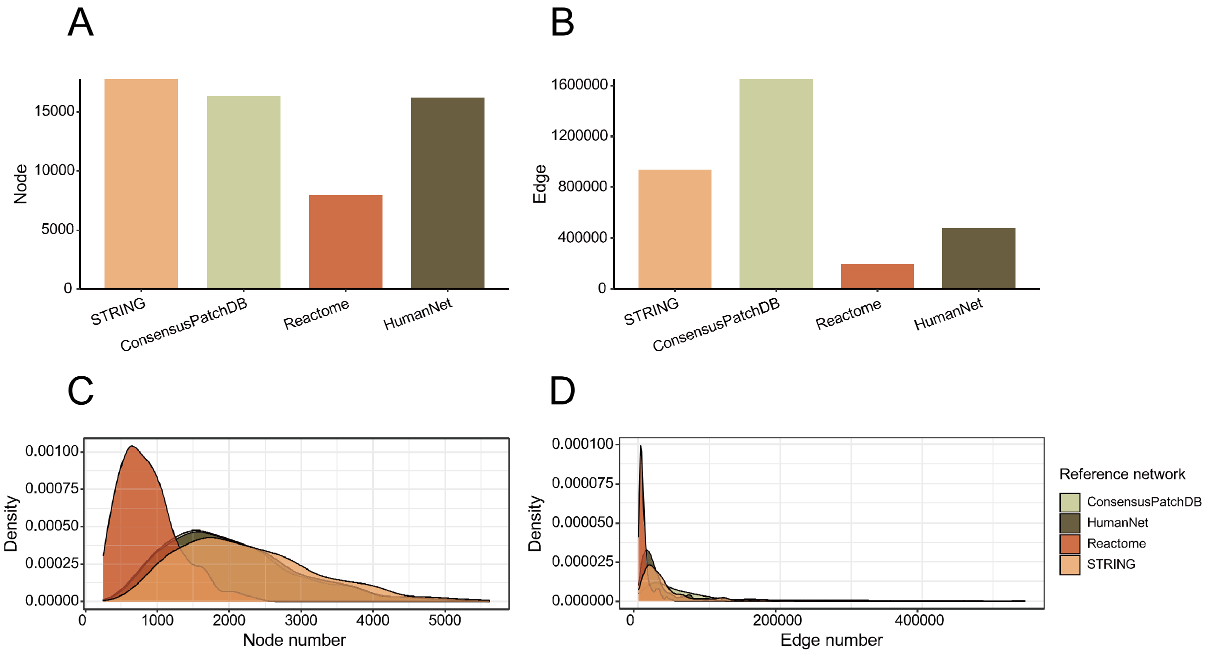

Figure S6. Statistics of cell-type-specific interactome networks guided by four different reference networks.

(A) The bar plot depicts the number of nodes in four different reference networks, including STRING, ConsensusPathDB, Reactome, and HumanNet.

(B) The bar plot depicts the number of edges in each reference network.

(C-D) Density plots show the number of nodes **(C)** and number of edges **(D)** in all cell-type-specific interactome networks inferred using four different reference networks.

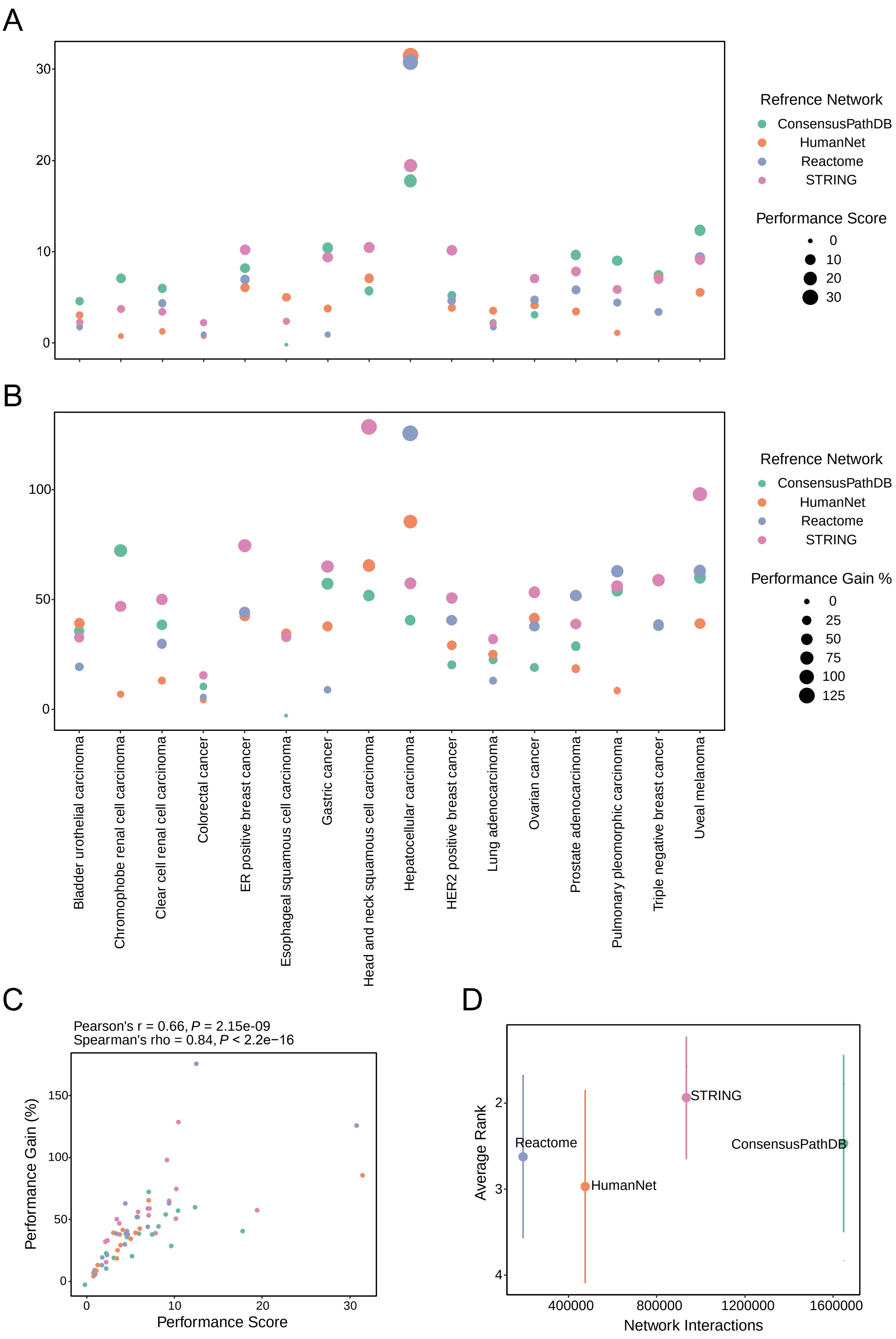
A
B
Refrence Network
ConsensusPathDB
HumanNet
Reactome
STRING
Performance Score
Performance Gain %
Bladder urothelial carcinoma
Chromophobe renal cell carcinoma
Clear cell renal cell carcinoma
Colorectal cancer
ER positive breast cancer
Esophageal squamous cell carcinoma
Gastric cancer
Head and neck squamous cell carcinoma
Hepatocellular carcinoma
HER2 positive breast cancer
Lung adenocarcinoma
Ovarian cancer
Prostate adenocarcinoma
Pulmonary pleomorphic carcinoma
Triple negative breast cancer
Uveal melanoma
C
Pearson's r = 0.66, P = 2.15e-09
Spearman's rho = 0.84, P < 2.2e−16
Performance Gain (%)
Performance Score
D
Average Rank
Network Interactions
STRING
Reactome
ConsensusPathDB
HumanNet

Figure S7. Network performance of recovering DisGeNET disease gene sets.

(A-B) The network performance score **(A)** and network performance gain **(B)** were evaluated on DisGeNET disease genes associated with specific tumor types in malignant networks. This analysis is conducted across 16 solid tumor types, where there is an overlap of more than 20 genes between the malignant network nodes and the disease genes of particular tumor type.

(C) The network performance gain was correlated with network performance score of malignant networks generated by four reference networks across 16 solid tumor types.

(D) The average ranked performance of malignant networks generated by each reference networks on the recovery of the DisGeNET disease genes compared with the number of interactions of reference networks. Error bars are one standard deviation of the average ranked network performances across each collection of recovery tasks.

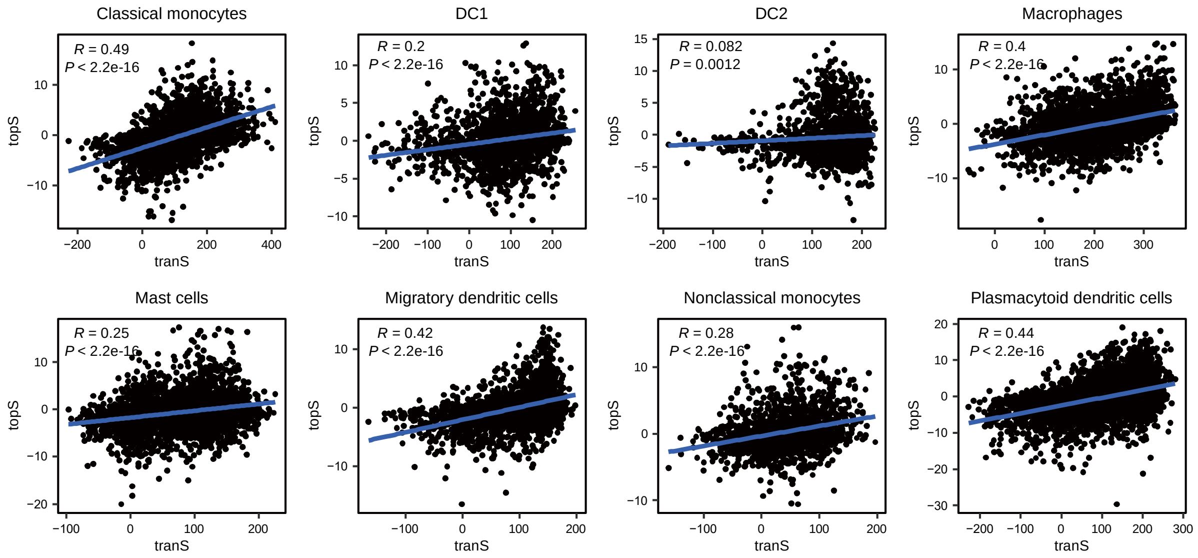

Figure S8. Comparison of topological specificity and transcriptional specificity scores in myeloid cells of pan-tumor TIMEs.

Scatter plots show corresponding topological and transcriptional specificity scores across genes of each myeloid subset-specific networks. Blue line represents the best linear regression fit.

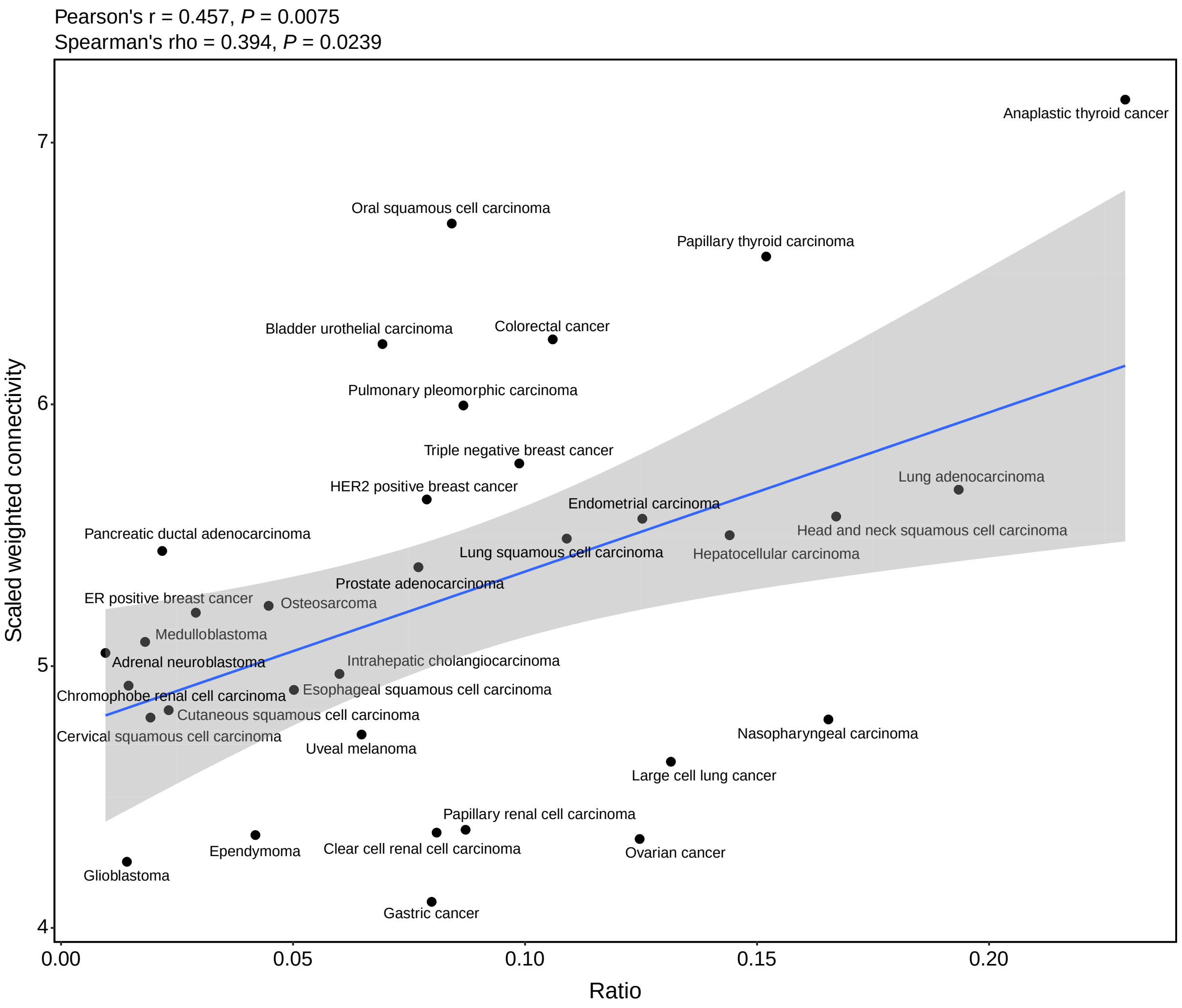

Figure S9. Connectivity of cytotoxic gene sets within *CD8*$^+$ T cell-specific networks across different tumor types.

The Scatter plot illustrates correlation between the fraction of *CD8*$^+$ T cells and the scaled weighted connectivity of cytotoxic genes in 33 solid tumor types. Blue line represents the best linear regression fit.

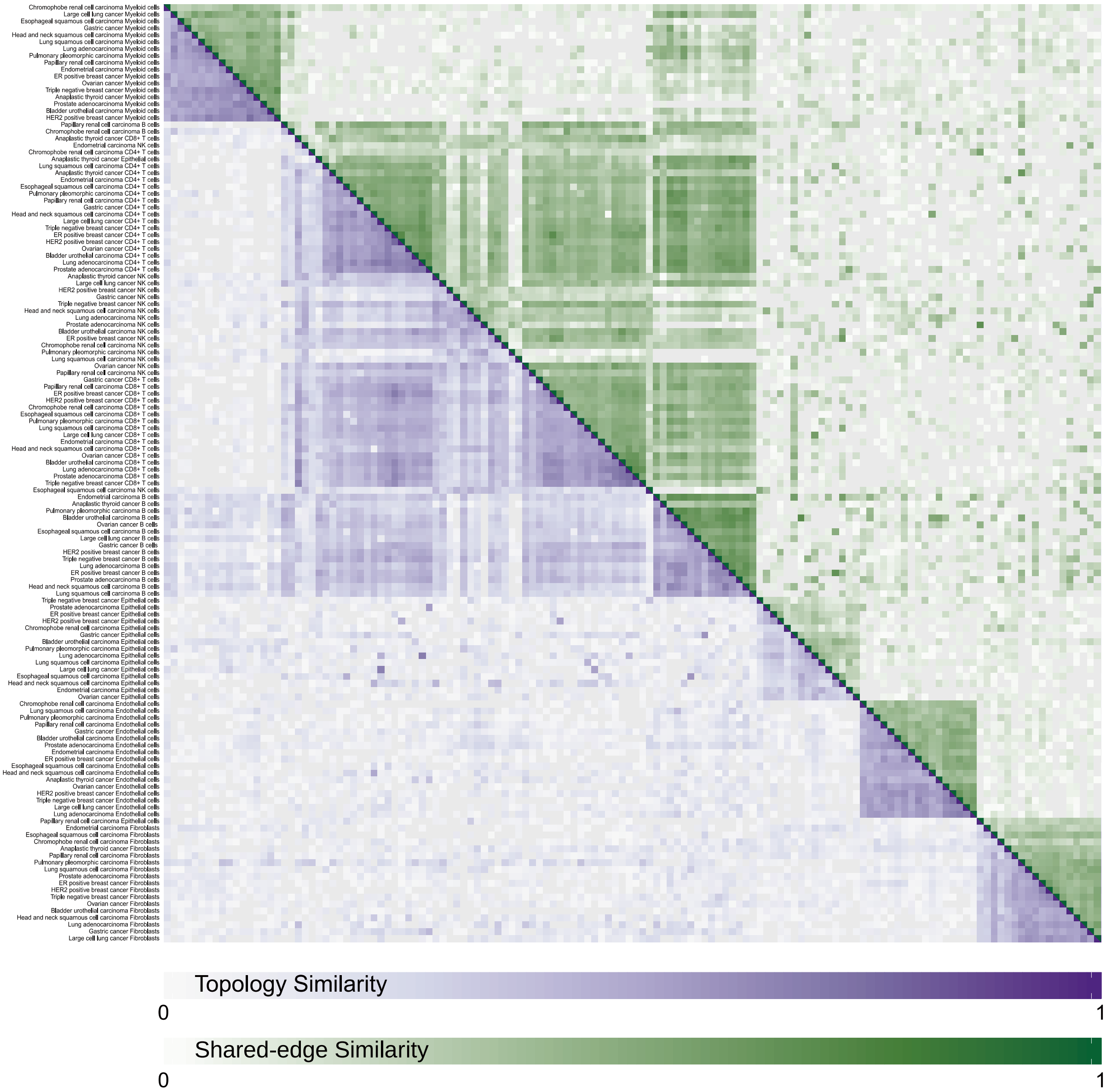

Figure S10. Network similarity across 17 solid tumor types.

The heatmap depicts the comparison of interactome networks specific to 8 cell types identified from 17 solid tumor types. The topology similarity and shared-edge similarity are calculated.

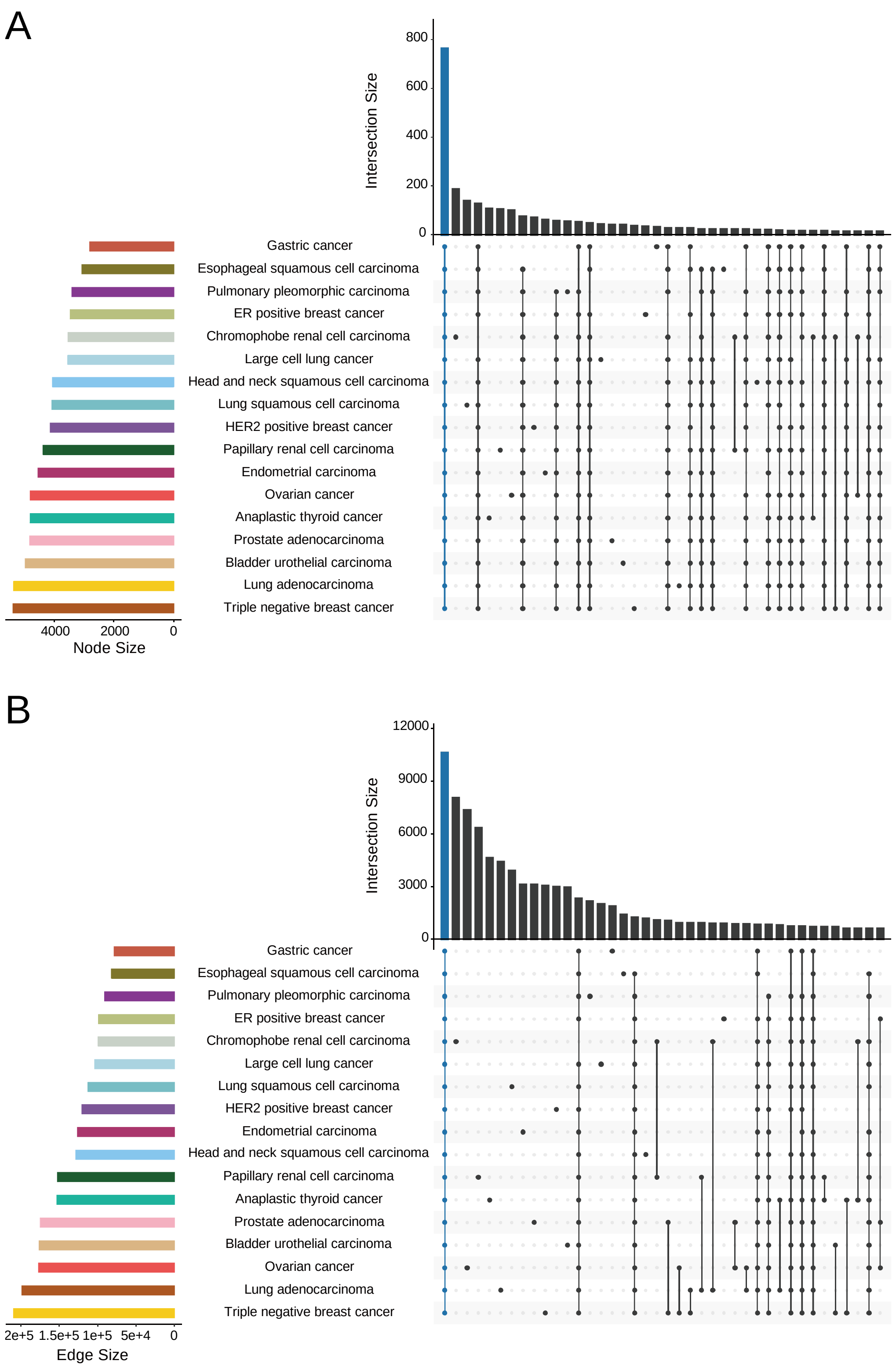

Figure S11. Comparison of *CD8*$^{+}$ T cells networks across 17 solid tumors.

(A-B) Upset plots show node **(A)** and edge **(B)** overlap of *CD8*$^{+}$ T cell-specific networks among 17 solid tumor types.

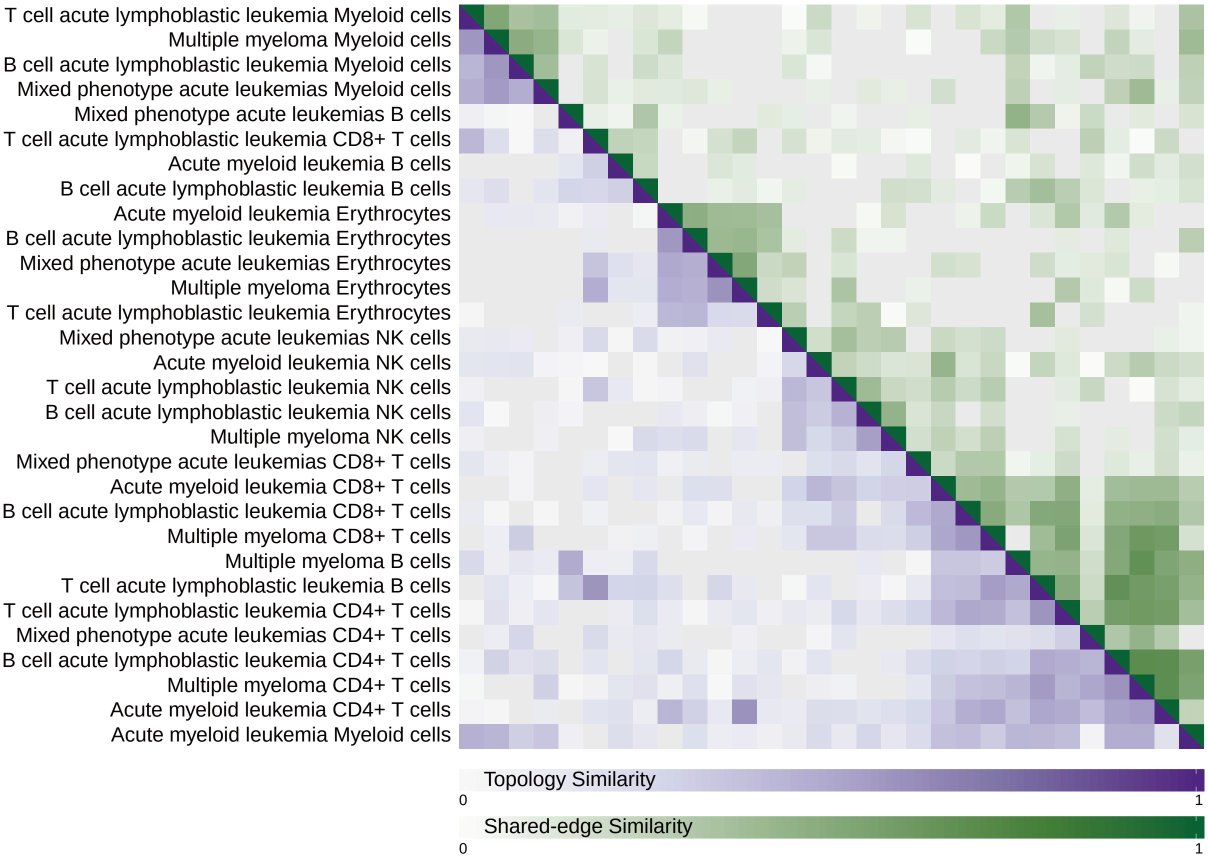

Figure S12. Network similarity across five hematologic malignancies.

The heatmap depicts the comparison of interactome networks specific to six cell types identified from five hematologic malignancies. The topology similarity and shared-edge similarity are calculated.

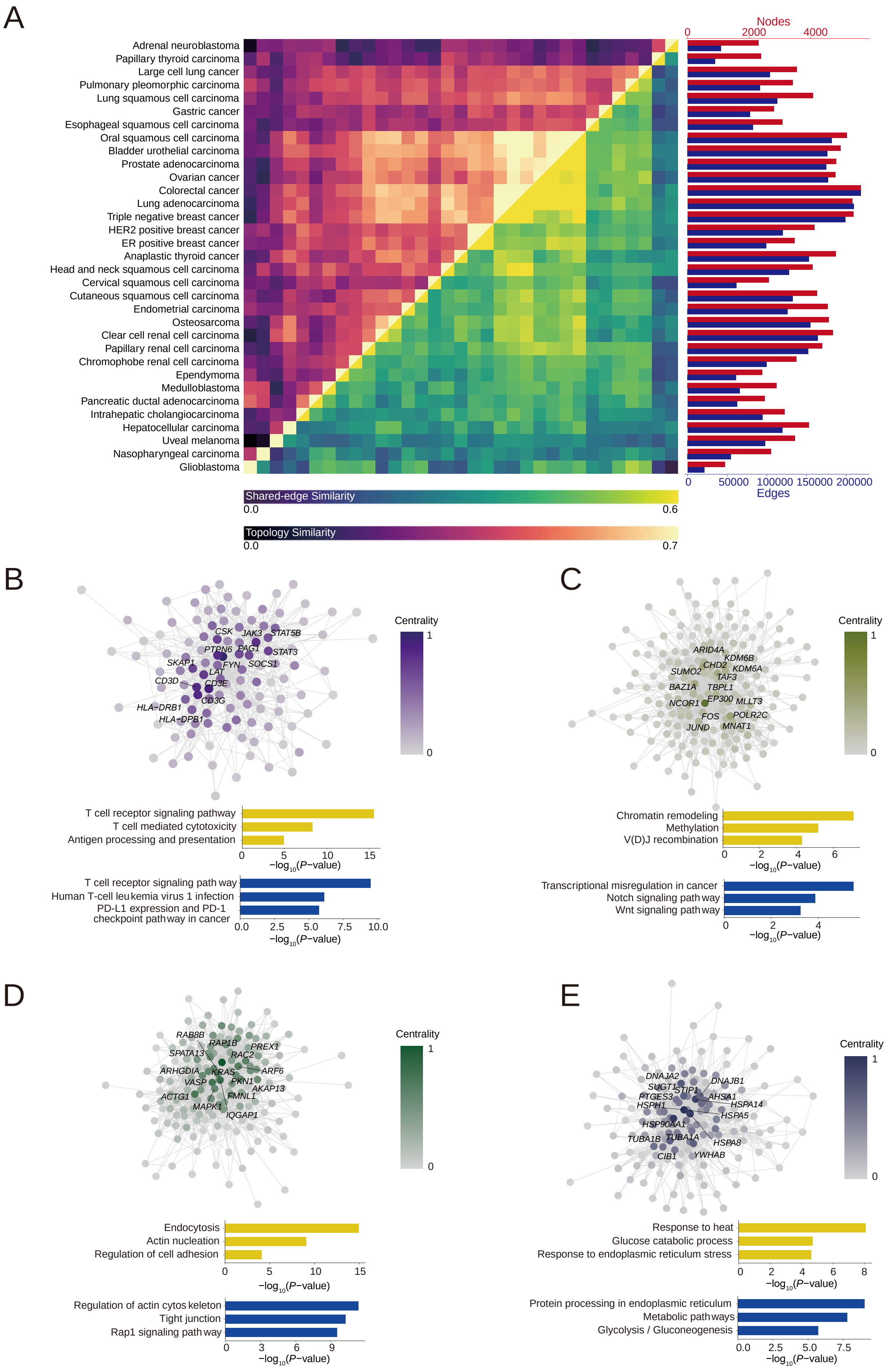
A
Nodes
0 2000 4000
Adrenal neuroblastoma
Papillary thyroid carcinoma
Large cell lung cancer
Pulmonary pleomorphic carcinoma
Lung squamous cell carcinoma
Gastric cancer
Esophageal squamous cell carcinoma
Oral squamous cell carcinoma
Bladder urothelial carcinoma
Prostate adenocarcinoma
Ovarian cancer
Colorectal cancer
Lung adenocarcinoma
Triple negative breast cancer
HER2 positive breast cancer
ER positive breast cancer
Anaplastic thyroid cancer
Head and neck squamous cell carcinoma
Cervical squamous cell carcinoma
Cutaneous squamous cell carcinoma
Endometrial carcinoma
Osteosarcoma
Clear cell renal cell carcinoma
Papillary renal cell carcinoma
Chromophobe renal cell carcinoma
Ependymoma
Medulloblastoma
Pancreatic ductal adenocarcinoma
Intrahepatic cholangiocarcinoma
Hepatocellular carcinoma
Uveal melanoma
Nasopharyngeal carcinoma
Glioblastoma
0 50000 100000 150000 200000
Edges
Shared-edge Similarity
0.0 0.6
Topology Similarity
0.0 0.7
B
Centrality
1
0
CSK JAK3 STAT5B
PTPN6 PAG1 STAT3
SKAP1 FYN SOCS1
LAT
CD3D CD3E
CD3G
HLA−DRB1
HLA−DPB1
T cell receptor signaling pathway
T cell mediated cytotoxicity
Antigen processing and presentation
0 5 10 15
−log10(P−value)
T cell receptor signaling path way
Human T-cell leukemia virus 1 infection
PD-L1 expression and PD-1 checkpoint pathway in cancer
0.0 2.5 5.0 7.5 10.0
−log10(P−value)
C
Centrality
1
0
ARID4A
KDM6B
CHD2 KDM6A
SUMO2
TAF3
BAZ1A TBPL1
NCOR1 EP300 MLLT3
FOS POLR2C
JUND MNAT1
Chromatin remodeling
Methylation
V(D)J recombination
0 2 4 6
−log10(P−value)
Transcriptional misregulation in cancer
Notch signaling path way
Wnt signaling path way
0 2 4
−log10(P−value)
D
Centrality
1
0
RAB8B
RAP1B PREX1
SPATA13 RAC2
ARHGDIA KRAS ARF6
VASP PKN1
AKAP13
ACTG1 FMNL1
MAPK1
IQGAP1
Endocytosis
Actin nucleation
Regulation of cell adhesion
0 5 10 15
−log10(P−value)
Regulation of actin cytos keleton
Tight junction
Rap1 signaling path way
0 3 6 9
−log10(P−value)
E
Centrality
1
0
DNAJA2
SUGT1 STIP1 DNAJB1
PTGES3 AHSA1
HSPH1 HSPA14
HSPA5
HSP90AA1
TUBA1B TUBA1A
HSPA8
CIB1 YWHAB
Response to heat
Glucose catabolic process
Response to endoplasmic reticulum stress
0 2 4 6 8
−log10(P−value)
Protein processing in endoplasmic reticulum
Metabolic path ways
Glycolysis / Gluconeogenesis
0.0 2.5 5.0 7.5
−log10(P−value)

Figure S13. Comparative analysis of interactome networks specific to *CD8*$^+$ T cells.

(A) The heatmap depicts the comparison of interactome networks specific to *CD8*$^+$ T cells identified from different tumor types. The red gradient panel represents the topology similarity estimated from shared nodes' topological specificity. The green gradient panel represents the edge similarity estimated from shared edges' interaction strengths. Network sizes are shown by number of nodes (red bars) and number of edges (blue bars).

(B-E) The graph plots depict four representative core subnetworks identified from the shared network of ER positive breast cancer and HER2 positive breast cancer. The centrality of each gene implicated in the subnetwork is labeled in color. The bar plots under each graph plot shows the GO (yellow bars) and KEGG (blue bars) pathway enriched for each core subnetwork.

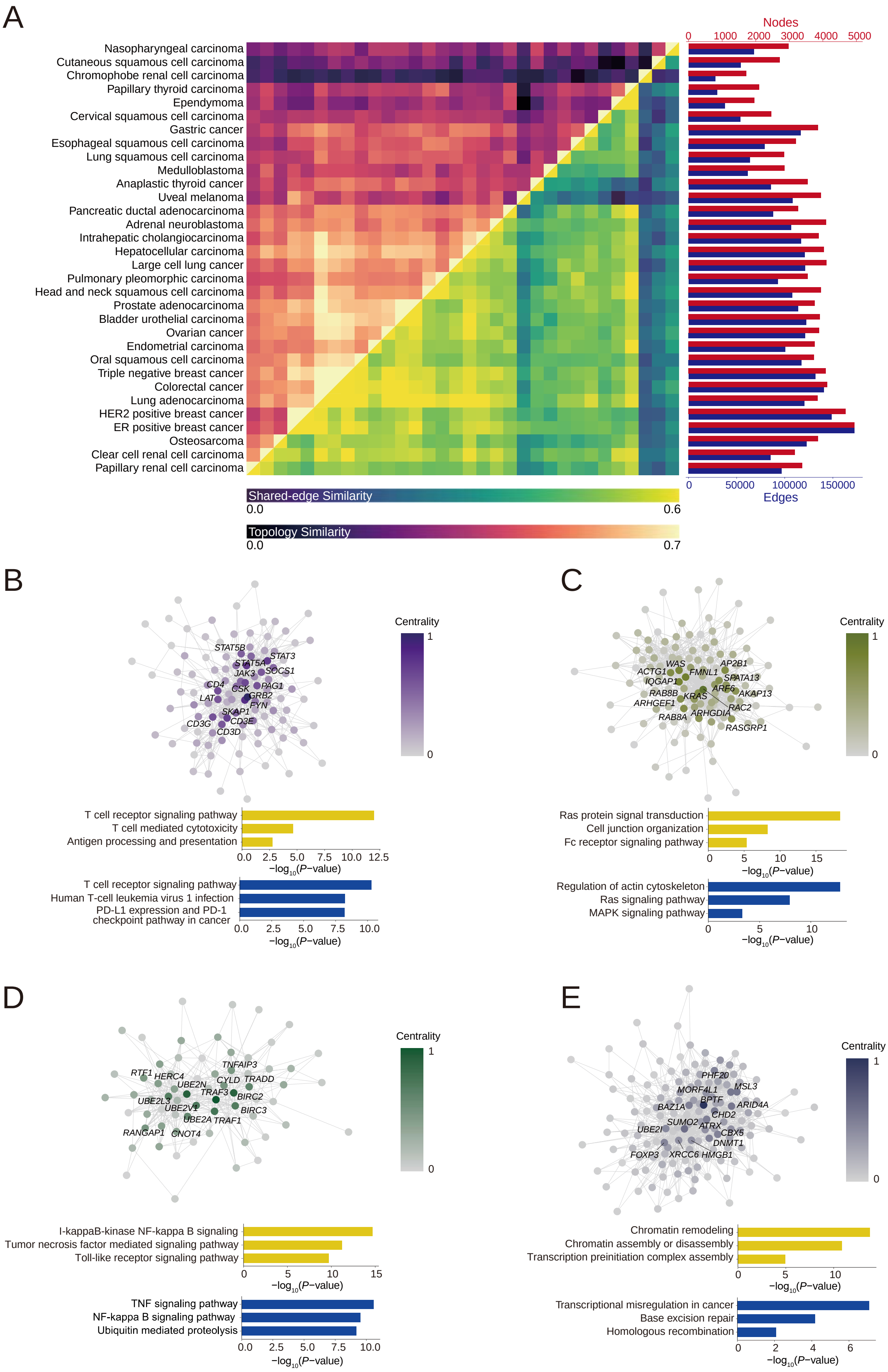
A
Nodes
0 1000 2000 3000 4000 5000
Nasopharyngeal carcinoma
Cutaneous squamous cell carcinoma
Chromophobe renal cell carcinoma
Papillary thyroid carcinoma
Ependymoma
Cervical squamous cell carcinoma
Gastric cancer
Esophageal squamous cell carcinoma
Lung squamous cell carcinoma
Medulloblastoma
Anaplastic thyroid cancer
Uveal melanoma
Pancreatic ductal adenocarcinoma
Adrenal neuroblastoma
Intrahepatic cholangiocarcinoma
Hepatocellular carcinoma
Large cell lung cancer
Pulmonary pleomorphic carcinoma
Head and neck squamous cell carcinoma
Prostate adenocarcinoma
Bladder urothelial carcinoma
Ovarian cancer
Endometrial carcinoma
Oral squamous cell carcinoma
Triple negative breast cancer
Colorectal cancer
Lung adenocarcinoma
HER2 positive breast cancer
ER positive breast cancer
Osteosarcoma
Clear cell renal cell carcinoma
Papillary renal cell carcinoma
0 50000 100000 150000
Edges
Shared-edge Similarity
0.0 0.6
Topology Similarity
0.0 0.7
B
Centrality
1
0
STAT5B STAT3 STAT5A SOCS1 JAK3 CD4 CSK PAG1 LAT GRB2 FYN SKAP1 CD3G CD3E CD3D
T cell receptor signaling pathway
T cell mediated cytotoxicity
Antigen processing and presentation
0.0 2.5 5.0 7.5 10.0 12.5
−log10(P−value)
T cell receptor signaling pathway
Human T-cell leukemia virus 1 infection
PD-L1 expression and PD-1 checkpoint pathway in cancer
0.0 2.5 5.0 7.5 10.0
−log10(P−value)
C
Centrality
1
0
WAS AP2B1 ACTG1 FMNL1 IQGAP1 SPATA13 ARF6 RAB8B AKAP13 KRAS ARHGEF1 RAC2 RAB8A ARHGDIA RASGRP1
Ras protein signal transduction
Cell junction organization
Fc receptor signaling pathway
0 5 10 15
−log10(P−value)
Regulation of actin cytoskeleton
Ras signaling pathway
MAPK signaling pathway
0 5 10
−log10(P−value)
D
Centrality
1
0
TNFAIP3 RTF1 HERC4 CYLD TRADD UBE2N TRAF3 BIRC2 UBE2L3 UBE2V1 BIRC3 UBE2A TRAF1 RANGAP1 CNOT4
I-kappaB-kinase NF-kappa B signaling
Tumor necrosis factor mediated signaling pathway
Toll-like receptor signaling pathway
0 5 10 15
−log10(P−value)
TNF signaling pathway
NF-kappa B signaling pathway
Ubiquitin mediated proteolysis
0.0 2.5 5.0 7.5 10.0
−log10(P−value)
E
Centrality
1
0
PHF20 MORF4L1 MSL3 BPTF BAZ1A ARID4A CHD2 SUMO2 UBE2I ATRX CBX5 DNMT1 FOXP3 XRCC6 HMGB1
Chromatin remodeling
Chromatin assembly or disassembly
Transcription preinitiation complex assembly
0 5 10
−log10(P−value)
Transcriptional misregulation in cancer
Base excision repair
Homologous recombination
0 2 4 6
−log10(P−value)

Figure S14. Comparative analysis of interactome networks specific to *CD4*$^{+}$ T cells.

(A) The heatmap depicts the comparison of interactome networks specific to *CD4*$^{+}$ T cells identified from different tumor types. The red gradient panel represents the topology similarity estimated from shared nodes' topological specificity. The green gradient panel represents the edge similarity estimated from shared edges' interaction strengths. Network sizes are shown by number of nodes (red bars) and number of edges (blue bars).

(B-E) The graph plots depict four representative core subnetworks identified from the shared network of bladder urothelial carcinoma and prostate adenocarcinoma. The centrality of each gene implicated in the subnetwork is labeled in color. The bar plots under each graph plot shows the GO (yellow bars) and KEGG (blue bars) pathway enriched for each core subnetwork.

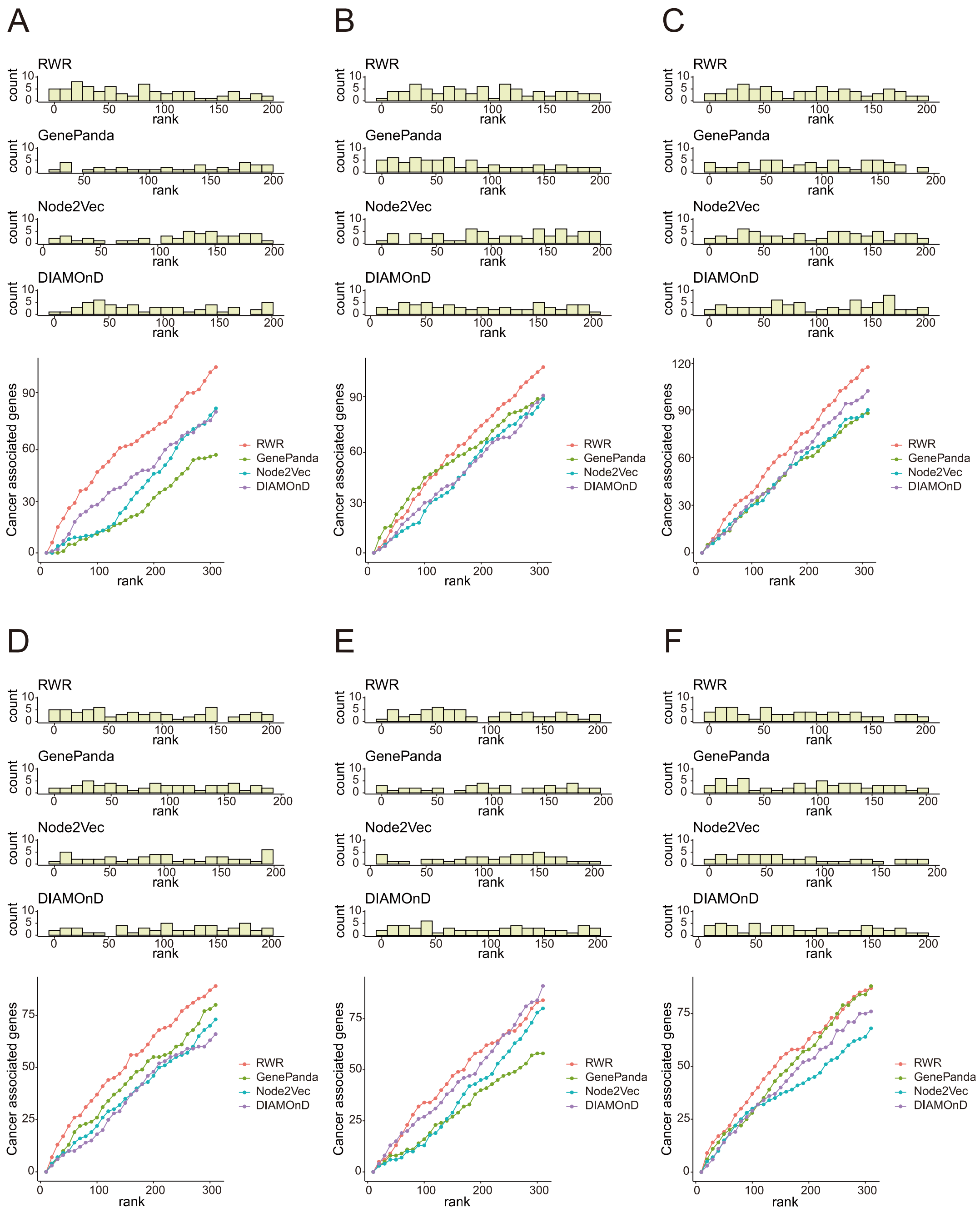

Figure S15. Comparing four network-based gene prioritization methods.

(A-F) Histograms show the distribution of overlap genes between top-ranked 200 genes and NCG genes used four gene prioritization methods in various malignant networks for acute myeloid leukemia **(A)**, B cell acute lymphoblastic leukemia **(B)**, chronic lymphocytic leukemia **(C)**, ER positive breast cancer **(D)**, papillary thyroid carcinoma **(E)** and uveal melanoma **(F)**. The line charts show the overlap genes size of top-ranked 300 genes (x-axis) and NCG genes (y-axis).

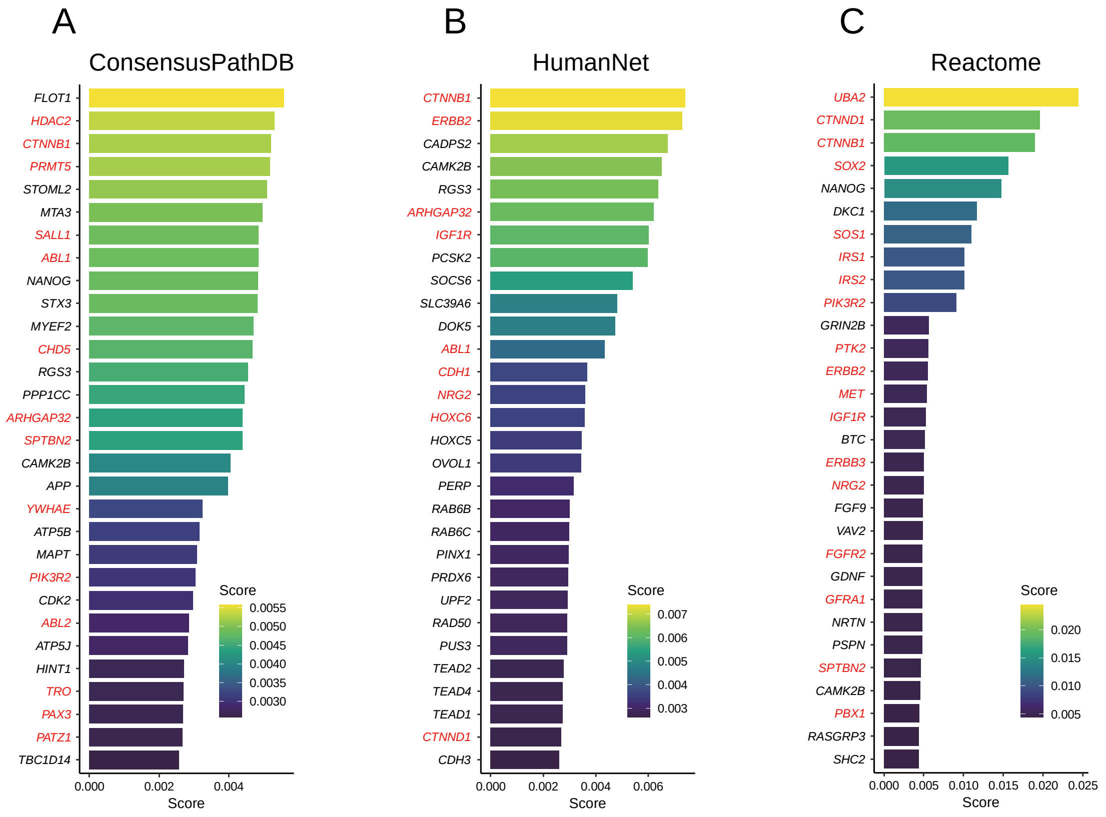

Figure S16. Prioritize risk genes in uveal melanoma.

In supplement to **Figure 6A**, this figure shows results of risk gene prioritization using the malignant melanocytes-specific interactome networks inferred from ConsensusPathDB **(A)**, HumanNet **(B)**, and Reactome **(C)**. The bar plots depict the stationary probability for each top-ranked gene from random walk with restart. The reported cancer driver genes in the NCG 6.0 database are labeled in red.

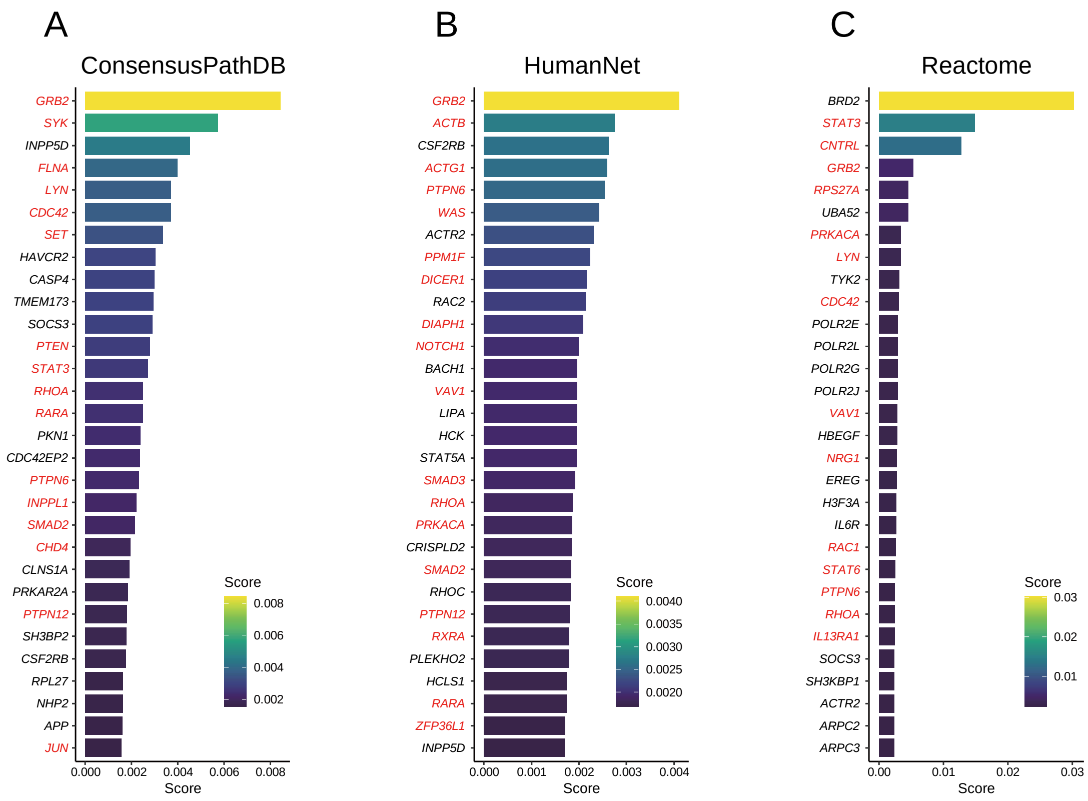

Figure S17. Prioritize risk genes in acute myeloid leukemia.

In supplement to **Figure 6B**, this figure shows results of risk gene prioritization using the malignant myeloid cell-specific interactome networks inferred from ConsensusPathDB **(A)**, HumanNet **(B)**, and Reactome **(C)**. The bar plots depict the stationary probability for each top-ranked gene from random walk with restart. The reported cancer driver genes in the NCG 6.0 database are labeled in red.

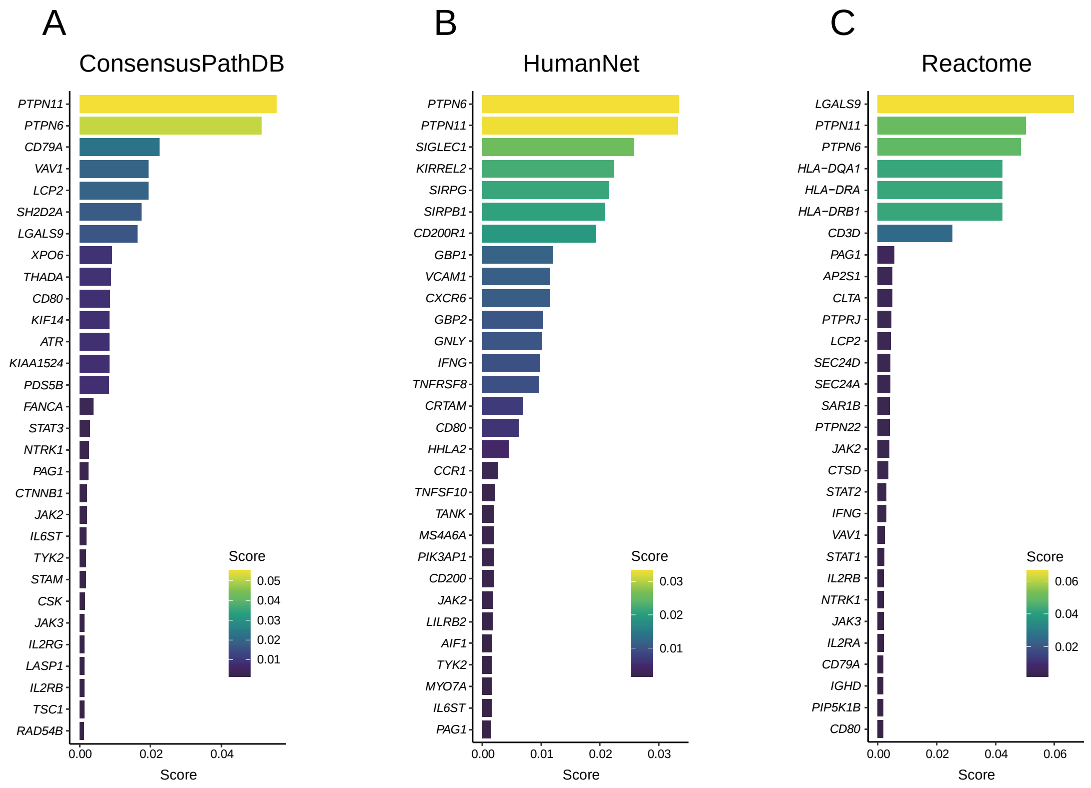

Figure S18. Prioritize risk genes for T cell exhaustion.

In supplement to **Figure 6C**, this figure shows the results of risk gene prioritization using the interactome networks specific to exhausted T cells in the pan-tumor TIMEs inferred from ConsensusPathDB **(A)**, HumanNet **(B)**, and Reactome **(C)**. The bar plots depict the stationary probability for each top-ranked gene from random walk with restart.

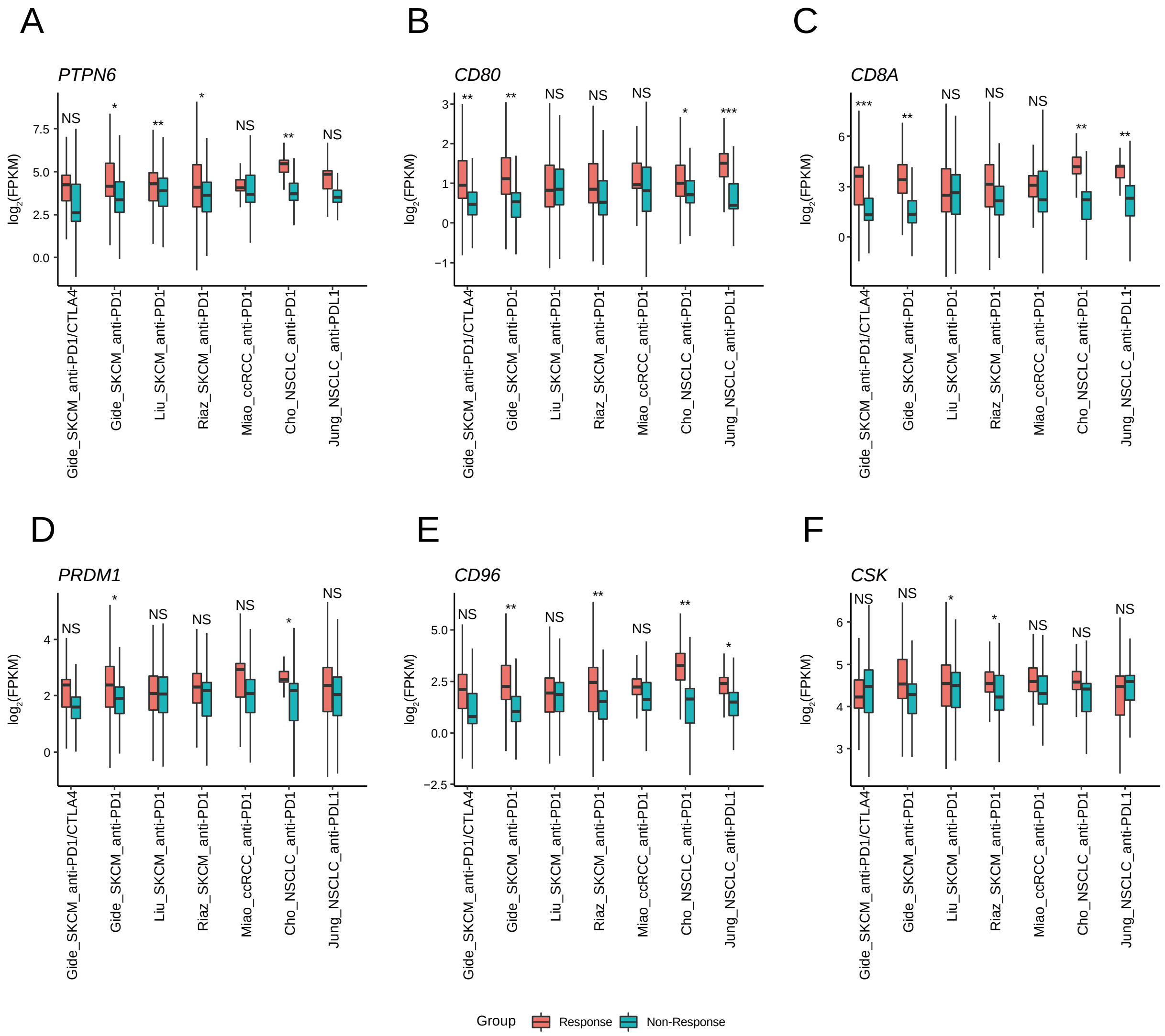

Figure S19. The expression levels of risk genes prioritized for T cell exhaustion in ICB cohorts.

The expression levels of *PTPN6* **(A)**, *CD80* **(B)**, *CD8A* **(C)**, *PRDM1* **(D)**, *CD96* **(E)**, and *CSK* **(F)** in six ICB therapy cohorts. The boxplots show the difference of their expression levels between ICB therapy response and non-response groups. The asterisks represent the degree of significance calculated using Limma (* *P*-value < 0.05, ** *P*-value < 0.01, *** *P*-value < 0.001).
